# Parp3 promotes astrocytic differentiation through a tight regulation of Nox4-induced ROS and mTorc2 activation

**DOI:** 10.1038/s41419-020-03167-5

**Published:** 2020-11-06

**Authors:** José-Manuel Rodriguez-Vargas, Kathline Martin-Hernandez, Wei Wang, Nicolas Kunath, Rajikala Suganthan, Jean-Christophe Amé, F. Javier Oliver, Jing Ye, Magnar Bjørås, Françoise Dantzer

**Affiliations:** 1 Poly(ADP-ribosyl)ation and Genome Integrity, Laboratoire d’Excellence Medalis, UMR7242, Centre Nationale de la Recherche Scientifique/Université de Strasbourg, Institut de Recherche de l’Ecole de Biotechnologie de Strasbourg, 300 bld. S. Brant, CS10413, 67412 Illkirch, France; 2 Department of Clinical and Molecular Medicine, Norwegian University of Science and Technology (NTNU), Trondheim, Norway; 3 Department of Microbiology, Oslo University Hospital and University of Oslo, Oslo, Norway; 4 Cellular Biology and Immunology, Instituto Lopez-Neyra de Parasitologia y Biomedicina, CSIC/IPBLN, CIBERONC, Parque Tecnologico de Ciencas de la Salud de Granada, 18016 Armilla, Spain

**Keywords:** Neuroscience, Stem-cell differentiation

## Abstract

Parp3 is a member of the Poly(ADP-ribose) polymerase (Parp) family that has been characterized for its functions in strand break repair, chromosomal rearrangements, mitotic segregation and tumor aggressiveness. Yet its physiological implications remain unknown. Here we report a central function of Parp3 in the regulation of redox homeostasis in continuous neurogenesis in mice. We show that the absence of Parp3 provokes Nox4-induced oxidative stress and defective mTorc2 activation leading to inefficient differentiation of post-natal neural stem/progenitor cells to astrocytes. The accumulation of ROS contributes to the decreased activity of mTorc2 as a result of an oxidation-induced and Fbxw7-mediated ubiquitination and degradation of Rictor. In vivo, mTorc2 signaling is compromised in the striatum of naïve post-natal *Parp3*-deficient mice and 6 h after acute hypoxia-ischemia. These findings reveal a physiological function of Parp3 in the tight regulation of striatal oxidative stress and mTorc2 during astrocytic differentiation and in the acute phase of hypoxia-ischemia.

## Introduction

In the developing and adult mammalian brain, continuous neurogenesis and brain plasticity depend on the sustained activity of neural stem/progenitor cells (NPSCs) which mainly reside in two neurogenic niches: the subventricular zone (SVZ) in the walls of the lateral ventricles and the subgranular zone (SGZ) of the dentate gyrus (DG) in the hippocampus. In both regions, NPSCs self-renew, migrate and retain the ability to differentiate into neurons, astrocytes and oligodendrocytes. Each step is tightly regulated by a variety of intrinsic and extrinsic molecular properties including the regulation of gene expression, morphogenic signaling pathways and cellular metabolism^1^. Among the many metabolic pathways that control NPSCs function and differentiation, the regulation of reactive oxygen species (ROS) production and the maintenance of an optimal redox state are central. Under physiological conditions, ROS act as important chemical modulators of signaling during neuronal development. In contrast abnormally increased levels of ROS have been linked to neuronal toxicity, aging and neurodegenerative diseases^2^.

Astrocytes are CNS-resident cells that beside providing metabolic and structural support and mediating neurotransmission are key in the control of redox homeostasis to secure nearby neuronal survival and function^3,4^. Unlike neurons, astrocytes generate high levels of mitochondrial ROS^5^. Moreover, an enzymatic source of ROS production through the activity of Nadph oxidases (Nox) has been largely documented in primary as well as stable astrocytic cultures^6–8^. Recently, the astrocytic mitochondrial ROS have been defined as pivotal regulators of brain metabolism and behavior^9^. However, during stress conditions in brain, such as cerebral ischemia, CNS inflammation and elevated levels of chemokines, high levels of ROS contribute to the dysfunction of astrocytes that consequently compromise tissue regeneration or aggravate neurotoxicity^10,11^. Thus, it is essential understanding the regulation of ROS production and the maintenance of redox homeostasis during neurogenesis and identifying the molecular players involved.

Oxidative damage in DNA is repaired primarily via the base excision repair pathway (BER). Functional disruption of enzymes that are involved in the processing of oxidative damage via the BER in mice have been causally associated with deficiencies in cognitive performance, in the resolution of neuroinflammation or in ischemic stroke recovery^12–15^.

Poly(ADP-ribose) polymerase 3 (PARP3) is a member of the PARP protein family that catalyzes mono-ADP-ribosylation (MARylation), the addition of a single ADP-ribose unit onto its protein or DNA targets. PARP3 has been characterized for its functions in the repair of DNA double-strand breaks via non homologous end-joining^16–19^, in the ADP-ribosylation of chromatin at site specific single strand breaks^20–22^, in chromosome rearrangements^23,24^, in mitotic segregation^17^ and in transcriptional regulation in the zebrafish^25^. More recently, PARP3 has also been defined for its contribution in tumor aggressiveness exemplifying its selective inhibition as an encouraging therapeutic strategy for chemo-resistant breast cancers^26,27^. However, whether and how PARP3 regulates physiological functions or failings has not been addressed yet. Here we identify and decipher a significant role of Parp3 in the modulation of Nox4-induced ROS that governs the activation of mTorc2 during differentiation of NPSC to astrocytes. In vivo, we show that the Parp3-mTorc2 axis is particularly important in the striatum of post-natal mice and shortly after hypoxia-ischemia.

## Materials and methods

### Animals and perinatal HI

The *Parp3*^*+/+*^ and *Parp3*^*–/–*^ mice have been previously described^17^. Cerebral hypoxia and ischemia were induced by permanent occlusion of the left common carotid artery (CCA) prior to systemic hypoxia as previously described^12^. In brief, P9 *Parp3*^*+/+*^ and *Parp3*^–^^*/–*^ mice were anesthetized with isoflurane (4% induction in the chamber followed by exposure to 2.5% Isoflurane maintenance equilibrated with an ambient of air and oxygen in the ratio 2:1) followed by skin incision at the anterior midline of the neck. Following artery preparation, a needle was placed into the artery and monopolar cauterization (Hyfrecator 2000; ConMed, Utica, NY USA) was carried out at a power of 4.0 W to electro coagulate the artery. Skin incisions were then closed by absorbable sutures (Safil 8-0, DRM6; B. Braun Melsungen Ag, Hessen, Germany). The entire operation, from skin incision to closure, lasted for approximately 5 min. Following a recovery period for 90 min the surgically treated mice were exposed to an hypoxic, humidified atmosphere containing 10% oxygen balance nitrogen (Yara, Oslo, Norway) for 60 min at 36.6 °C. The pups were returned to their mother and after 6 h the brains were retrieved and prepared for immunohistochemistry or striatum excision. Sham-treated animals were subjected to anesthesia, skin incision with suturing, but not CCA occlusion and hypoxia.

### Neurosphere cultures, differentiation, knockdown experiments and treatments

To generate independent NPSCs, forebrains of *Parp3*^*+/+*^ and *Parp3*^*–/*^^–^ at postnatal day 8 were dissected and mechanically dissociated following incubation in 0.05% Trypsin solution containing 0.02% EDTA for 10 min at 37 °C. Isolated cells were filtered through a 70 µm cell strainer and resuspended in a growing serum-free Neurobasal Medium (Gibco) supplemented with 1% N2, 2% B27 supplements (Gibco), 2 mM Glutamax (Gibco), 20 ng ml^−1^ basic fibroblastic growth factor (bFGF, Gibco), 10 ng ml^−1^ human epithelial growth factor (hEGF, Gibco) and 1% Penicillin/Streptomycin. To form neurospheres, cells were grown on ultralow adherent Nunclon Sphera Dishes (ThermoFisher). For culture expansion, neurospheres were collected every 7 days, dissociated with Accutase Cell Detachment Solution (Corning) at 37 °C for 10 min. Single cell suspensions were replated at a dilution of 1/5 and maintained for several passages. For astrocyte differentiation assays, viable single cells were seeded at a density of 3.5 × 10^6^ cells 10 ml^−1^ (P100 dishes) or 6 × 10^5^ cells 3 ml^−1^ (6 well/plates) in plates coated with Poly-l-Lysine and Laminin (Sigma-Aldrich). Differentiation was induced with Dulbecco’s Modified Eagle’s Medium (DMEM) High Glucose 4.5 g l^−1^ supplemented with 1% N2 supplement (Invitrogen), 2 mM GlutaMAX (Invitrogen), 1% fetal bovine serum (FBS) and 1% Penicillin/Streptomycin. Medium was renewed every 2 days for up to 12 days. For knockdown experiments, viable single cells were plated at a density of 3×10^6^ cells 10 ml^−1^ in the growing Neurobasal medium as above for two days before transfection. Cells were transfected with 50 nM siRNA using the Amaxa NucleoFector 4D protocol for stem cells in suspension following the manufacturer’s instructions (Lonza). Gene-specific siRNAs (ON_TARGET plus smart pool) for FBXW7 (J-04153), NOX4 (J-058509), NF-kB p65^RelA^ (L-040776) and the negative control siRNA (D-001810) were obtained from Dharmacon (Thermo Fisher Scientific). Cells were processed for the indicated experiments from 48 h to 72 h later. For treatment with the chemical agents, viable single cells were seeded at 1.5 × 10^6^ cells ml^−1^ 24 h before treatment. Cells were treated with CoCl_2_ (Sigma) or Deferoxamine Mesylate Salt (DFM, Sigma) at the indicated concentrations for 24 h. Medium was renewed and cells were processed as indicated.

### Cell and tissue extracts and immunoblotting

For total extracts, cells (3 × 10^6^ cells 10 ml^−1^ P100) were washed twice in cold PBS1x containing 0.5 mM Pefabloc (Roche) and lysed by incubation on ice for 20 min in RIPA-like buffer (50 mM Tris-HCl pH8, 1% Triton X-100, 0.25% Na Deoxycholate, 150 mM NaCl, 1 mM EDTA, 50 mM NaF, 20 mM Na Pyrophosphate, 1 mM Na Orthovanadate, 1 mM Pefabloc (Roche), 1X protease inhibitor cocktail (Roche). After centrifugation at 13,000 rpm at 4 °C for 15 min, cleared suspension was quantified by Bradford Protein Assay (Biorad). For nuclear extracts, cells (3.5 × 10^6^ cells 10 ml^−1^) were scraped in cold PBS1x containing 0.5 mM Pefabloc and centrifuged at 1600 rpm for 5 min. The pellets were resuspended by Dounce homogenization in hypotonic buffer containing 10 mM Tris-HCl pH 7.3, 10 mM KCl, 1.5 mM MgCl_2_, 10 mM β-mercaptoethanol and 0.2 mM phenylmethanesulfonyl fluoride (PMSF) on ice. After centrifugation at 2000g for 5 min at 4 °C, the pellets were resuspended in extraction buffer containing 15 mM Tris-HCl pH 7.3, 0.4 M NaCl, 1 mM EDTA, 1 mM MgCl_2_, 10% Glycerol, 10 mM β-mercaptoethanol and 0.2 mM PMSF. Samples were incubated 30 min on ice and centrifuged at 16,000 *g* for 30 min at 4 °C. The supernatant was used as the nuclear extract fraction and quantified by Bradford Protein Assay (Biorad). When processing brain tissues, tissue biopsies (10–100 mg) were collected from the *Parp3*^*+/+*^ and *Parp3*^*–/*^^–^ mice and stored in liquid nitrogen until further analyses. The biopsies were fragmented using a scalpel and immediately lysed and homogenized in a highly denaturing guanidine-isothiocyanate-containing buffer to isolate intact DNA, RNA and proteins (AllPrep DNA/RNA/Protein Mini Kit, Qiagen). Proteins were resolved on 10% or 5% SDS-PAGE polyacrylamide gels and Mini-PROTEAN^R^TGX^TM^ Stain-Free Gels 4–12% (Biorad) and transferred onto PVDF Membrane (Biorad, Berkeley, CA). The blots were blocked with 5% semi-skimmed milk powder in PBS1x containing 0.1% Tween-20 for 60 min and incubated overnight with the appropriate primary antibodies (Supplementary Table 1) followed by incubation with the appropriate horseradish peroxidase-conjugated antibodies. Proteins were detected using ECL-PRIME (GE Healthcare) and the imaging system Image Quant LAS 4000 (GE Healthcare). Bands were analyzed by densitometry using Image J software (NIH).

### Immunoprecipitation experiments

Equivalent amounts of RIPA-like NSPC cell extracts (2 mg) were diluted in dilution buffer DB (20 mM Tris-HCl pH7.5, 0.1% NP40, 150 mM NaCl, 1 mM Pefabloc) and pre-cleared by incubation on protein A/G Sepharose beads for 1 h at 4 °C before incubation with the indicated antibodies (Supplementary Table 1) overnight at 4 °C followed by 2 h incubation at 4 °C with protein A/G Sepharose (GE Healthcare, Little Chalfont, UK). Beads were washed twice with DB containing 250 mM NaCl and twice with DB containing 150 mM NaCl. Beads were then resuspended in Laemmli buffer and analyzed by SDS-PAGE and immunoblotting as above.

### Immunohistochemistry

For immunostaining of mouse brain (P9) with anti p-GSK3β (S9) and anti p-AKT (S473) we used paraffin-embedded samples, cut at 4 µm thickness. Antigen-retrieval was done using a combined pH 6.0 and heat approach. Antibody incubation was done overnight at 4 °C. Pictures were imaged using a confocal microscope (Zeiss LSM880, Jena, Germany) at 10x magnification and a 20 plane z-stack. Regions of interest were dissected digitally after 3D-rendering in Imaris 9.3 (Bitplane, Oxford Instruments, Zurich, Switzerland). Every image was dissected by hand, the region was adapted dynamically whenever needed due to shifted anterior-posterior sampling position. The Allen Mouse Brain Atlas (right side, http://mouse.brain-map.org/static/atlas) served as a reference. We measured intensity levels as a correlate of immunohistochemical reactivity levels in the ipsilateral relative to the contralateral side of the hypoxic-ischemic event. The quotient of both absolute intensity values was used as the “relative” level.

### Immunofluorescence microscopy

For immunostaining cells were seeded on coated glass cover-slips at a density of 6×10^5^ cells 3 ml^−1^ in 6-well plates and processed for astrocytic differentiation as described above. Cells were fixed with 3% paraformaldehyde, PBS1x solution for 15 min at 25 °C. Fixed cells were permeabilized with 0.1% Triton-X100, PBS1x solution for 15 min at 25 °C and blocked in PBS1x containing 5% BSA, 5% Goat Serum, 0.1% Triton-X100 for 1 h at 25 °C. Cells were incubated with the mouse anti-GFAP antibody (Supplementary Table 1) overnight at 4 °C followed by incubation with a fluorescent-conjugated secondary antibody for 3 h at 25 °C in a humid atmosphere. Cells were mounted on microscope slides with DAPI-Fluoromount mounting medium for fluorescence (Southern Biotech) and analyzed using a Leica CTR MIC Confocal Microscope.

### RNA sequencing and RT-qPCR analysis

Total RNA from cells was isolated using the RNAeasy kit (Qiagen) according to the manufacturer’s protocol. For RNAseq, total RNA-Seq libraries were generated from 500 ng of total RNA using TruSeq Stranded Total RNA LT Sample Prep Kit with Ribo-Zero Gold (Illumina, San Diego, CA) as detailed in Supplementary Information. Sequencing was performed on an Illumina HiSeq 4000 in a 1x50bp single end format as detailed in Supplementary Information.

For RT-qPCR, RNA was processed for reverse transcription using the Maxima Enzyme MIX cDNA reverse transcription kit (ThermoFisher) according to the manufacturer’s instructions. Real time PCR was performed using the QuantiTect SYBRGreen PCR Kit (Quiagen Quality) combined with the StepOne plus Real Time PCR detection systems (Applied Biosystems) according to the manufacturer’s protocol. The PCR products were analyzed with the StepOne Software. The quantity of PCR products was estimated by the relative standard curve method and the ΔΔCt method. Samples were analyzed in triplicates and normalized using the *GAPDH* or *βactin* housekeeping genes as indicated. The primer sequences used for qPCR are listed in Supplementary Table 2.

### ROS production and superoxide production

Single NSPC cells were seeded at 5 × 10^5^ cells 3 ml^−1^ in 6-well plates and processed for differentiation to astrocytes as detailed above. At the indicated time points, cells were washed twice with sterile PBS1x and maintained in Hank’s balanced Salt Solution (Thermo Fisher Scientific). ROS were measured immediately using the fluorescent dye-based free radical sensor carboxy-H_2_DCFDA (FLUKA) according to the manufacturer’s instructions combined with spectrometry using a Nanoquant Infinite M200 TECAN Instrument. Fresh medium and cells without probe were used as controls. Mitochondrial superoxide were measured using the MitoSox Red mitochondrial superoxide fluorescent indicator according to the manufacturer’s protocol. The absorbance was measured using a FLA9500 GE Typhoon Biomolecular Imager with a specific laser Ex/Em 540/580 nm. H_2_O_2_ 10 mM for 10 min was used as a positive control of mitochondrial superoxide production. All experiments were performed in three independent biological replicates with 2 technical replicates for each.

### In vivo ubiquitination assay

NSPC were seeded at a density of 3 × 10^6^ cells per P100 plates. 24 h after plating, the cells were transfected with 5 µg of HA-Ubiquitin (Addgene) following the Amaxa NucleoFector 4D protocol for stem cells in suspension (Lonza). Cells were maintained in culture for 48 h, mock-treated or treated with MG132 (1 µM, Sigma) for 6 h and lysed using the RIPA-like buffer as above. Equivalent amounts of total protein extracts (2 mg) were processed for immunoprecipitation and immunoblotting as detailed above using the appropriate antibodies (Supplementary Table 1).

### Dimedone labeling and analysis of oxidized Rictor

NSPC were treated with 10 mM dimedone (5,5-dimehtyl-1,3-cyclohexanedione, Sigma-Aldrich) for 2 h and lysed using the RIPA-like buffer as above. Equivalent amounts of total protein extracts (2 mg) were processed for control and Rictor immunoprecipitation using the appropriate antibodies. Oxidized proteins were detected using an anti-cysteine sulfenic acid antibody that detects the content of oxidized cysteines.

### Statistical analysis

Unless otherwise indicated, all experiments were performed using 3 independent isolations of *Parp3*^*+/+*^ and *Parp3*^–^^*/–*^ NPSC and three independent repeated experiments were performed. The data were expressed as mean values of the triplicates ± s.d. GraphPad Prism (Version 5, CA, USA) or R softwares were used to perform statistical analysis. Parametric data were analyzed using a two-tailed, unpaired Student’s *t*-test. A *P-*value < 0.05 was considered statistically significant for all comparisons.

## Results

### Parp3 deficiency causes incomplete differentiation of NPSCs to astrocytes

**Fig. 1:**
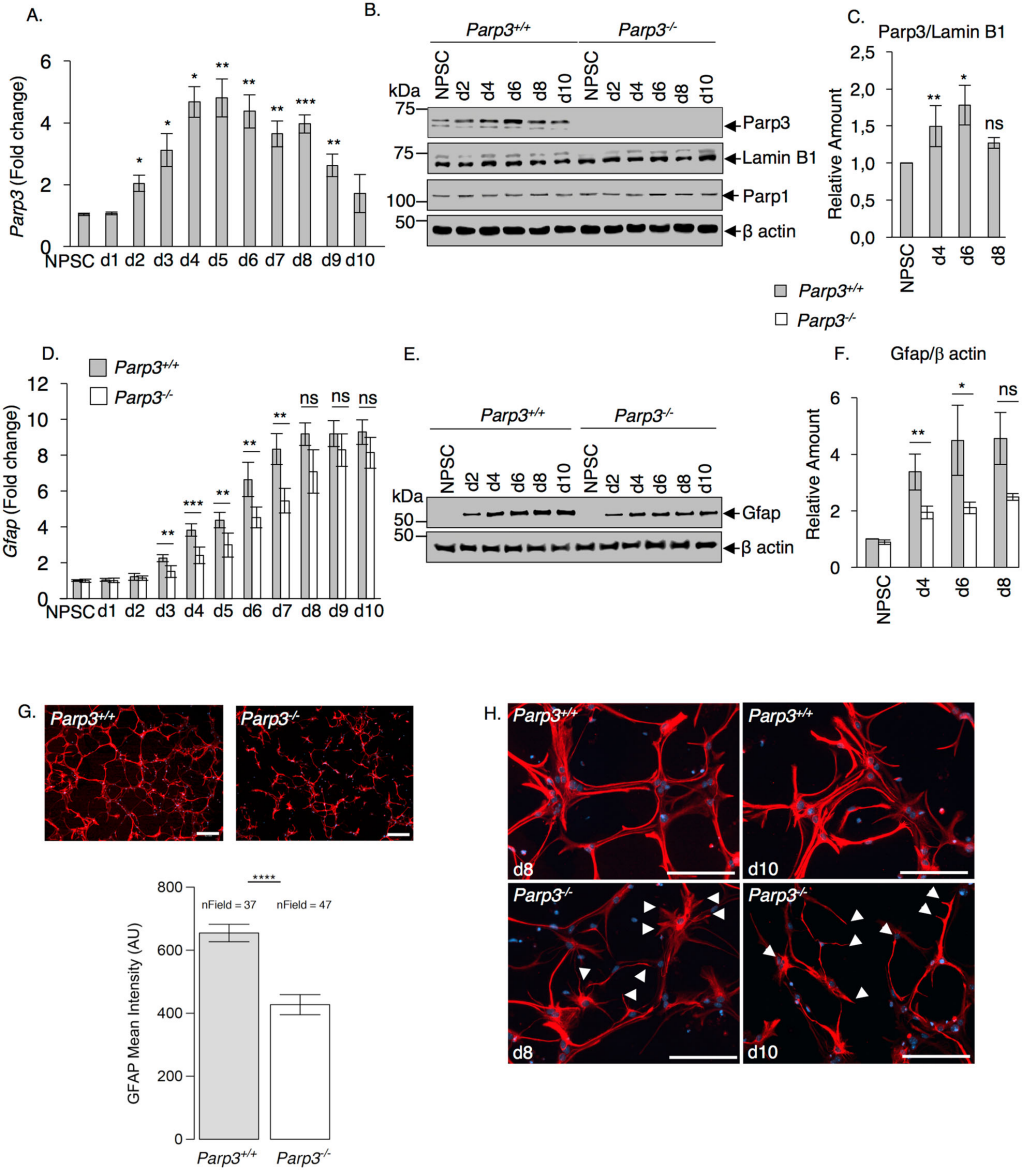
Parp3 is required for efficient astrocyte differentiation. **a** qPCR for *Parp3* in proliferating NPSCs and throughout differentiation to astrocytes (d1 to d10). Data are expressed relative to *Gapdh*. Values represent means ± s.d of three independent experiments and the three independent clones. *P < 0.05, **P < 0.01, ***P < 0.001. **b** Western blot analysis for Parp3, and Lamin B1 as control, Parp1 and β actin as control in proliferating NPSCs and throughout differentiation to astrocytes in nuclear extracts from *Parp3*^*+/+*^ and *Parp3*^*–/*^^–^ cultures. *Parp3* levels increase throughout differentiation (d2-d10) to peak at days d4-d6 and decrease again when differentiation completes. The expression levels of Parp1 remain constant throughout differentiation in *Parp3*^*+/+*^ and *Parp3*^*–/*^^–^ cultures. **c** The bar graph depicts the relative signal intensities of Parp3 relative to Lamin B1 in *Parp3*^*+/+*^ cultures using Image J. Values represent means ± s.d. of three independent experiments and two independent clones. *P < 0.05, **P < 0.01. ns, non-significant (*P* = 0.111). **d** qPCR for *Gfap* in proliferating NPSCs and throughout differentiation to astrocytes (d1-d10) in *Parp3*^*+/+*^ and *Parp3*^*–/*^^–^ cultures. Data are expressed relative to *Gapdh*. Values represent means ± s.d. of three independent experiments and ≥2 independent clones. **P < 0.01, ***P < 0.001, ns, non-significant (*P* = 0.059 d8, *P* = 0.061 d9, *P* = 0.101 d10). **e** Western blot analysis for Gfap relative to β actin in total extracts of *Parp3*^*+/+*^ and *Parp3*^*–/*^^–^ NPSCs and throughout glial differentiation (d2-d10). **f** The histogram depicts the relative signal intensities of Gfap relative to β actin in *Parp3*^*+/+*^ and *Parp3*^*–/*^^–^ cultures using Image J. Values represent means ± s.d. of three independent experiments and two independent clones. *P < 0.05, **P < 0.01, ns, non-significant (*P* = 0.051). **g** *Upper panel:* Representative immunofluorescence images of Gfap+ astrocytes in *Parp3*^*+/+*^ and *Parp3*^*–/*^^–^ cultures at d8 of differentiation. Scale bars, 0.2 μm. *Lower panel*: Quantification of the total intensity of Gfap labeling in *Parp3*^*+/+*^ versus *Parp3*^*–/*^^–^ astrocytes d8. Values represent means ±s.d. of three independent experiments and two independent clones. ****P < 0.0001. **h** High magnification images of Gfap+ astrocytes in *Parp3*^*+/+*^ and *Parp3*^*–/*^^–^ cultures at d8 and d10 of differentiation. White arrows point onto immature and unconnected ramifications detected in the Parp3^*–/*^^–^ astrocytes, revealing an imperfect interconnected network. Scale bars, 0.2 μm.

**Fig. 2:**
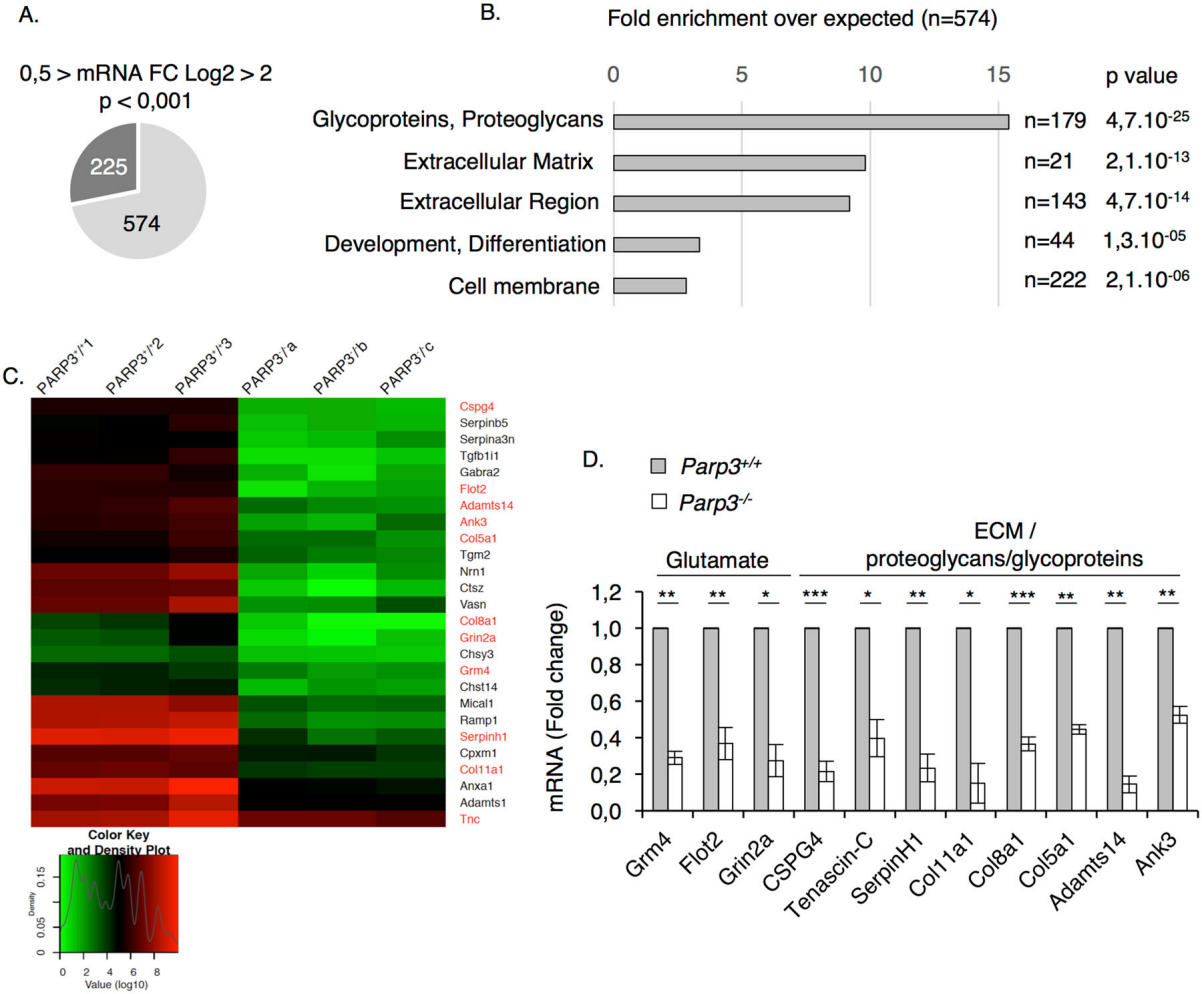
RNA seq reveals downregulation of transcripts coding for proteoglycans and proteins of the ECM in *Parp3*-deficient astrocytes. **a** Venn diagram illustrating the number of deregulated transcripts in *Parp3*^*–/*^^–^ versus *Parp3*^*+/+*^ astrocytes d4 (574 downregulated genes and 225 upregulated genes). **b** Pathway overrepresentation analysis of the 574 downregulated transcripts deduced with the DAVID bioinformatics resources 6.8. Number in brackets indicate the number of genes enriched, clustering to the corresponding pathway. **c** Clustered heatmap of downregulated transcripts in the ECM, membranes, glycoproteins and proteoglycans clusters. Transcripts highlighted in red were used for subsequent validation experiments. **d** qPCR validation of the selected downregulated transcripts in *Parp3*^*+/+*^ and *Parp3*^*–/*^^–^ astrocytes d4. Data are expressed relative to *Gapdh*. Error bars represent s.e.m. of three independent experiments. *P < 0.05, **P < 0.01, ***P < 0.001.

To investigate cell-intrinsic properties of Parp3 in neurogenesis, we used the neurosphere assay to quantify the capacity of NPSC to form multipotent clonal aggregates and to differentiate into glial lineage^28,29^. NPSCs isolated from the brain of *Parp3*^–^^*/–*^ post-natal mice were not affected in their ability to form primary neurospheres and they displayed normal levels of proliferation and self-renewal compared to *Parp3*^*+/+*^ NPSCs (Supplementary Fig. 1). To next explore the impact of Parp3 deficiency on NPSC differentiation capacity, we first monitored the mRNA and protein expression profile of Parp3 throughout NPSC differentiation to astrocytes (Fig. 1a–c). The mRNA and protein expression levels of Parp3 were moderate in the proliferating NPSCs, but increased markedly from d2 of differentiation peaking from d3 to d7 upon differentiation potentially indicating a contribution of Parp3 in astrocytic differentiation. Parp3 expression levels then gradually declined when differentiation terminated. We then analyzed the effect of Parp3 loss on differentiation of NPSCs to the glial lineage in vitro. Single cell suspensions of primary or secondary *Parp3*^*+/+*^ and *Parp3*^*–/*^^–^ neurospheres were induced to differentiate and astrocytes were identified following the expression of Gfap (Fig. 1d–f). Compared to the wild-type NPSCs, Parp3-deficient NPSCs displayed reduced mRNA and protein expression of Gfap suggesting an altered capacity to differentiate to astrocytes. Moreover, reproducible obvious signs of compromised morphology were observed in the Parp3-deficient astrocytes. While the *Parp3*^*+/+*^ astrocytes displayed ramified Gfap positive networks with extensive arborization and branching characterizing reactive astrocytes, the *Parp3*^*–/*^^–^ astrocytes displayed an immature, atrophied-like morphology with weaker Gfap staining (Fig. 1g, h and Supplementary Fig. 2a). However, the viability of the astrocytes was not affected (Supplementary Fig. 2b). To further decipher the molecular mechanism and biological pathways underlying this phenotype, RNA seq of the *Parp3*^*+/+*^ and *Parp3*^*–/*^^–^ astrocytes were performed 4-days upon differentiation. A pathway overrepresentation analysis of the 574 downregulated transcripts using the DAVID interface revealed substantial changes in pathways and molecules associated with the extracellular matrix and space or membranes including proteoglycans, and glycoproteins that are usually produced by reactive astrocytes and determine their development and astrocytic response^30,31^ (Fig. 2a, b). A clustered heatmap of a panel of selected transcripts from these groups and the validation of some genes by qPCR analysis confirmed that the absence of Parp3 significantly downregulated the selected transcripts consistently in three independent experiments (Fig. 2c, d). Notably, a majority of these transcripts are associated with synapse development and function (*Nrn, Gabra2, Ank3, Grin2a*) and/or receptor activity (*Grm4, Flot2, Grin2a*) and/or cell adhesion (*Flot2, Ank3, Col8a1*). These findings indicate that Parp3 contributes to the differentiation of NPSC towards the astroglial lineage and positively controls the biological properties of reactive astrocytes.

### Parp3 loss-induced differentiation deficits is caused by enhanced Nox4-dependent ROS production

Appropriate timing and completion of astrocyte differentiation is governed by a highly regulated tempo-spatial production of endogenous Reactive Oxygen Species (ROS)^32,33^. Moreover, in numerous pathophysiological models, ECM homeostasis and composition, synapse formation and activity, and cell adhesion are influenced by the levels of ROS^34–37^. We have previously reported a function of PARP3 in the cell response to ROS^27^. Thus, we surmised that the compromised astrocyte differentiation in the *Parp3*^*–/*^^–^ cells can be caused by an imbalanced redox metabolism. To validate this hypothesis, we first compared the sensitivity of the *Parp3*^*+/+*^ and *Parp3*^*–/*^^–^ NPSCs to ROS-generating chemicals by analyzing the impact on neurosphere formation and proliferation (Supplementary Fig. 3). *Parp3*^*–/−*^ NPSCs displayed a significantly reduced capacity to form neurospheres when exposed to paraquat, menadione and H_2_O_2_ compared to the *Parp3*^*+/+*^ NPSCs, and their proliferation rate was significantly reduced, supporting a role of Parp3 in cell response to oxidative stress in NPSCs.

**Fig. 3:**
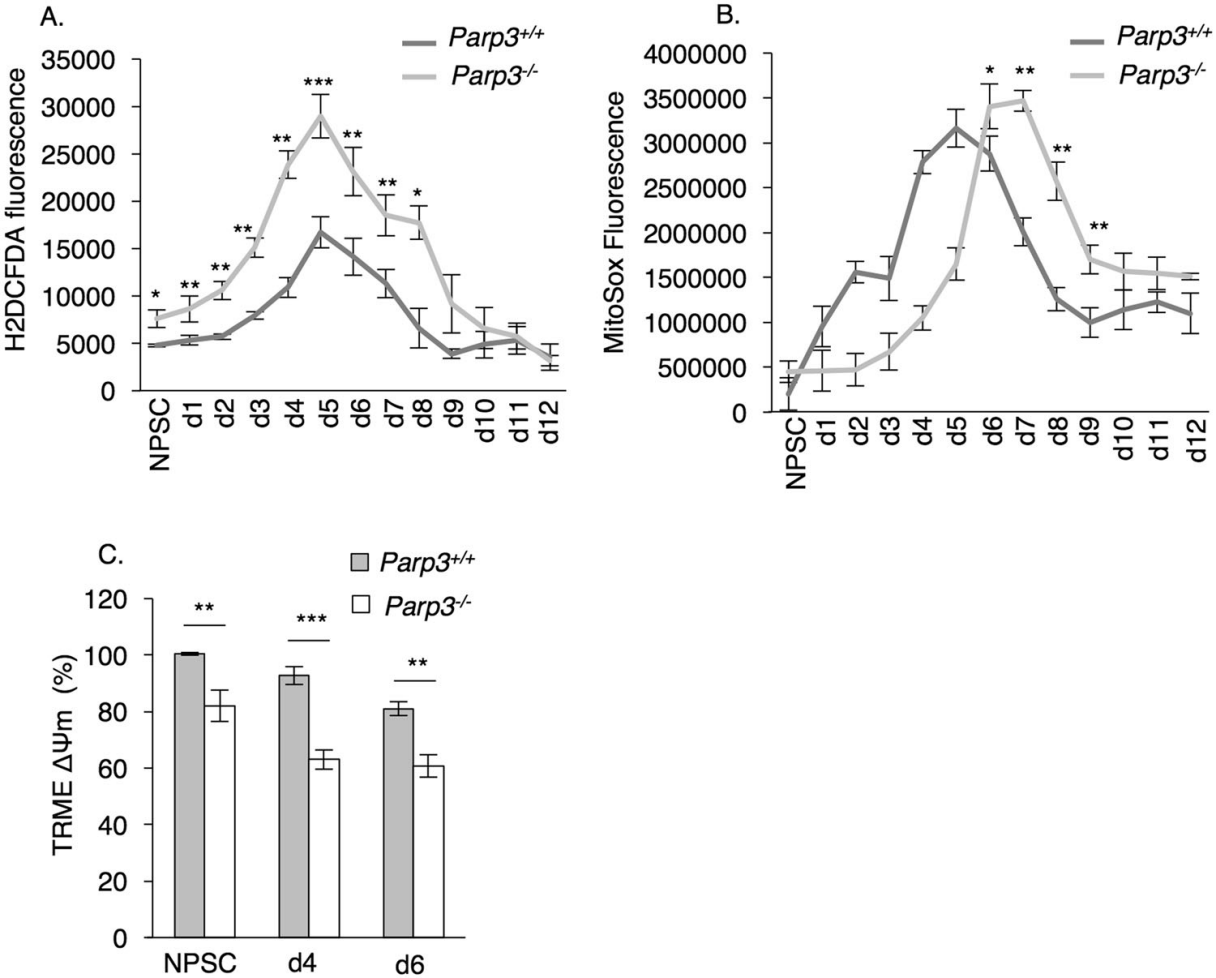
The absence of Parp3 causes oxidative stress and mitochondrial dysfunction in NPSC and astrocytes. **a** Measurement of total ROS production in NPSCs and throughout astrocyte differentiation (d1-d12) in *Parp3*^*+/+*^ versus *Parp3*^*–/*^^–^ cultures. Values represent means ± s.d. of three biological replicates and two independent clones. *P < 0.05, **P < 0.01, ***P < 0.001. **b** Measurement of mitochondrial ROS production in NPSCs and throughout differentiation (d1-d12) in *Parp3*^*+/+*^ versus *Parp3*^*–/*^^–^ cultures. Values represent means ± s.d. of three biological replicates and two independent clones. *P < 0.05, **P < 0.01. **c** Measurement of mitochondrial membrane potential in NPSC and astrocytes (d4, d6) in *Parp3*^*+/+*^ versus *Parp3*^*–/*^^–^ cultures. Values represent means ± s.d. of three biological replicates and two independent clones. **P < 0.01, ***P < 0.001.

Next, we addressed the role of Parp3 in modulating the redox status throughout differentiation to the astroglial lineage. *Parp3*^*+/+*^ and *Parp3*^*–/*^^–^ NPSCs were differentiated to astrocytes and the level of endogenous ROS production was determined using the fluorescent dye-based free radical sensor H_2_DCFDA (Fig. 3a). During glial differentiation in wild type cells, the production of ROS followed the expression of Parp3, by increasing gradually from d1 to d6 and decreasing again when differentiation terminated. While Parp3-deficient cells displayed a similar profile, the levels of ROS produced was significantly higher throughout differentiation indicating that Parp3 prevents excessive generation of ROS. One of the main contributors to ROS are the mitochondria. We used the MitoSOX Red mitochondrial superoxide indicator to stain *Parp3*^*+/+*^ and *Parp3*^*–/*^^–^ NPSCs throughout astrocytic differentiation (Fig. 3b). Mitochondrial ROS peaked at d5 during wild-type astrocytic differentiation, while ROS in *Parp3*^*–/*^^–^ cells peaked significantly later (d6-7) suggesting dysfunctional mitochondria. In support of this, the mitochondrial transmembrane potential was significantly reduced in the Parp3-deficient NPSC and astrocytes (Fig. 3c). These results suggest that Parp3 is involved in regulation of mitochondrial ROS.

**Fig. 4:**
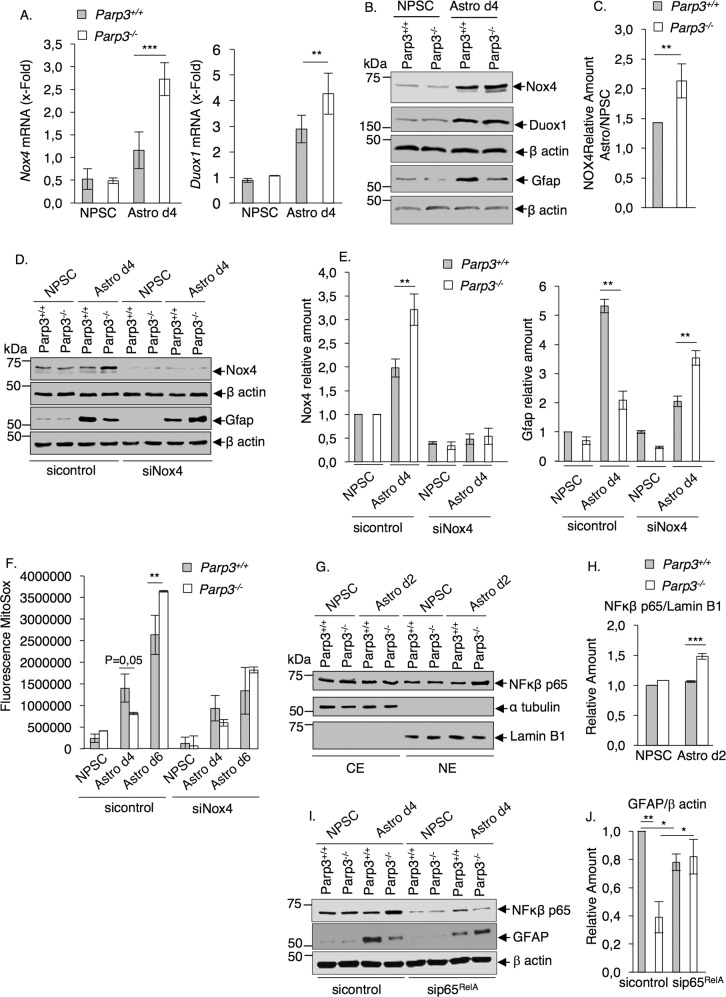
Increased Nox4 levels in Parp3-deficient astrocytes cause uncomplete differentiation. **a** qPCR for *Nox4* and *Duox1* Nadph oxidases in *Parp3*^*+/+*^ and *Parp3*^*–/*^^–^ NPSCs and d4 astrocytes. Data are expressed relative to *Gapdh*. Values represent the means ±s.e.m. of three independent experiments and two independent clones. **P < 0.01, ***P < 0.001. **b** Western blot analysis for the levels of Nox4 and Duox1 relative to β actin in *Parp3*^*+/+*^ and *Parp3*^*–/*^^–^ NPSCs and d4 astrocytes. The expression of Gfap relative to β actin confirms impaired astrocyte differentiation in the *Parp3*^*–/*^^–^ versus the *Parp3*^*+/+*^ cultures. **c** The bar graph depicts the relative fold increase of Nox4 levels in astrocytes relative to NPSCs in the *Parp3*^*+/+*^ and *Parp3*^*–/*^^–^ genotypes. Values represent means ± s.d. of three independent experiments and two independent clones. **P < 0.01. **d** The depletion of Nox4 re-establishes astrocyte differentiation in the *Parp3*^*–/*^^–^ cultures. Western blot analysis for the expression levels of Nox4 relative to β actin and Gfap relative to β actin in sicontrol and siNox4-treated *Parp3*^*+/+*^ and *Parp3*^*–/*^^–^ NPSCs and astrocytes d4. **e** The bar graph depicts the fold increase in the expression of Nox4 (left) and Gfap (right) in the Nox4 silenced (siNox4) relative to the control (sicontrol) *Parp3*^*+/+*^ and *Parp3*^*–/*^^–^ NPSC and astrocytes d4. Values represent means ± s.d. of three independent experiments. **P < 0.01. **f** The depletion of Nox4 restaures the increased levels of mitochondrial ROS observed in the *Parp3*^*–/*^^–^ astrocytes d6 to the levels detected in Parp3^+/+^ astrocytes d6. Measurements of mitochondrial ROS production in sicontrol and siNOX4-treated *Parp3*^*+/+*^ versus *Parp3*^*–/*^^–^ NPSC and astrocytes (d4, d6). Values represent means ± s.d. of three biological replicates. **P < 0.01. **g** *Parp3*^*–/*^^–^ astrocytes display enhanced nuclear translocation of NF-κB p65. Western blot analysis for the levels of NF-κB p65 subunit in cytoplasmic (CE) and nuclear (NE) extracts of *Parp3*^*+/+*^ and *Parp3*^*–/*^^–^ NPSCs and astrocytes d2. Lamin B1 and *α* tubulin were used as loading controls of NE and CE, respectively. **h** The bar graph depicts the fold increase in the levels of NF-κB p65 in NE versus CE and relative to *Parp3*^*+/+*^ NPSC set to 1. Values represent means ± s.d. of three independent experiments. ***P < 0.001. **i** The depletion of NF-kB p65 restores the expression of GFAP in the *Parp3*^*–/*^^–^ astrocytes d4 ssuggesting improved differentiation. Western blot analysis for the expression levels of NF-kB p65 and GFAP relative to β actin in sicontrol and sip65^RelA^-treated *Parp3*^*+/+*^ and *Parp3*^*–/*^^–^ NPSCs and astrocytes d4. **j** The bar graph depicts the fold increase in the levels of GFAP relative to β actin in sicontrol and sip65^RelA^-treated *Parp3*^*+/+*^ and *Parp3*^*–/*^^–^ astrocytes d4 and relative to *Parp3*^*+/+*^ sicontrol set to 1. Values represent means ± s.d. of three independent experiments. **P < 0.01; *P < 0.05.

In various organs including the central nervous system, the mitochondrial NADPH oxidase (Nox4) is a major source of ROS production^38–40^. Moreover, fine-tune regulation of the levels of Nox4-induced ROS is prime for efficient differentiation^41–43^. Thus, searching for a mechanism by which Parp3 regulates the redox balance, we analyzed the effect of Parp3 loss on the expression levels of Nox4 and Duox1 as control (Fig. 4a–c and Supplementary Fig. 4). The differentiation of *Parp3*^*+/+*^ NPSCs into astrocytes induced an upregulation of Nox4 at mRNA and protein levels. This increase was further enhanced in the *Parp3*^*–/*^^–^ astrocytes. In contrast, while an increased upregulation of *Duox1* was also detected in the *Parp3*^*–/*^^–^ astrocytes compared to the *Parp3*^*+/+*^ astrocytes, the protein expression levels remained comparable (Supplementary Fig. 4). Thus, the excessive production of ROS in the Parp3-deficient astrocytes may be explained by an apparent increase in the levels of Nox4. We therefore examined the impact of Nox4 depletion on the capacity of *Parp3*^*+/+*^ and *Parp3*^*–/*^^–^ NPSC to differentiate to astrocytes by analyzing the levels of Gfap (Fig. 4d, e). The silencing of Nox4 reduced the capacity of the *Parp3*^*+/+*^ NPSCs to differentiate to astrocytes supporting the notion that Nox4 activity contributes to astroglial differentiation. In contrast the silencing of Nox4 in *Parp3*^*–/*^^–^ NPSCs restored their capacity to differentiate to astrocytes. These results reinforce the hypothesis that the impaired differentiation of *Parp3*^*–/*^^–^ astrocytes is partly caused by enhanced levels of Nox4 and mitochondrial oxidative stress. In support of this, the enhanced levels of mitochondrial ROS detected in the *Parp3*^*–/*^^–^ astrocytes d6 were restored to the levels of the *Parp3*^*+/+*^ astrocytes d6 upon Nox4 silencing (Fig. 4f).

NF-kB is an essential regulator of Nox4 expression and Nox4-derived ROS production in muscle cells^44^. We have previously identified a role of PARP3 in the TG2-Snail-E cadherin axis in cancer progression, a signaling pathway driven by NF-kB^27^. To explain further the mechanism of Nox4 upregulation in the *Parp3*^*–/*^^–^ cells, we analyzed the nuclear translocation of NF-kB p65 as a marker of its activation in the *Parp3*^*+/+*^ and *Parp3*^*–/*^^–^ NPSCs and astrocytes (Fig. 4g, h). There was no significant change in cytoplasmic versus nuclear distribution of NF-kB p65 in the *Parp3*^*–/*^^–^ NPSCs versus the control *Parp3*^*+/+*^ NPSCs but the *Parp3*^*–/*^^–^ astrocytes displayed an increase in the nuclear translocation of NF-kB p65 compared to the *Parp3*^*+/+*^ astrocytes. In support of this, the depletion of p65^RelA^, restored the levels of Gfap in *Parp3*^*–/*^^–^ astrocytes d4 (Fig. 4i, j). These data indicate that Parp3 modulates NF-kB-mediated Nox4 expression for efficient differentiation.

### Parp3 deficiency and increased Nox4-dependent ROS production cause impaired mTorc2 pathway activation during astrocyte differentiation

**Fig. 5:**
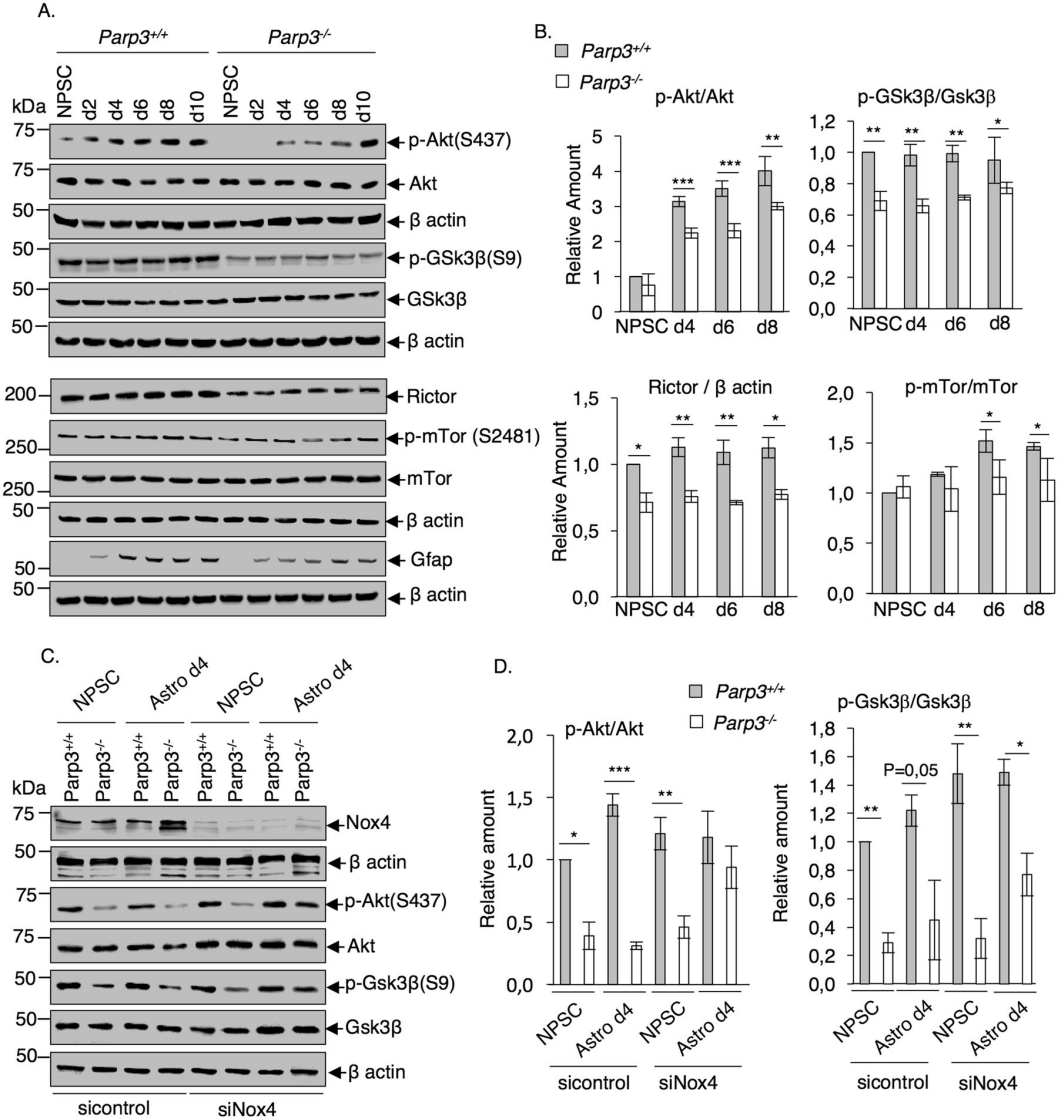
Increased Nox4 levels in Parp3-deficient astrocytes cause impaired activation of mTorc2. **a** Western blot analysis for the levels of p-Akt (S473), Akt and β actin as loading control, p-Gsk3β (S9), Gsk3β and β actin as loading control, Rictor, p-mTOR(S2481), mTOR and β actin as loading control and GFAP relative to β actin as loading control in NPSCs and throughout astrocytes differentiation (d2-d10) in *Parp3*^*+/+*^ and *Parp3*^*–/*^^–^ cultures. **b** Bar graphs depict the relative signal intensities of p-Akt versus Akt, p-Gsk3β versus Gsk3β, Rictor versus β actin and p-mTor versus mTor measured in three independent experiments and two independent clones using Image J. Mean values ± s.d. are indicated. *P < 0.05, **P < 0.01, ***P < 0.001. **c** The depletion of Nox4 re-establishes p-Akt(S473) expression in Parp3-deficient astrocytes. Western blot analysis for the expression of Nox4 versus β actin as loading control, p-Akt (S473) versus Akt and p-Gsk3β versus Gsk3β and β actin used as loading control in sicontrol (siCTL) and siNox4-treated *Parp3*^*+/+*^ and *Parp3*^*–/*^^–^ NPSCs and astrocytes d4. **d** Bar graphs depict the relative signal intensities of p-Akt versus Akt, p-Gsk3β versus Gsk3β, measured in three independent experiments using Image J. Values represent means ± s.d. of three independent experiments. *P < 0.05, **P < 0.01, ***P < 0.001.

In cancer cells, ROS production is coupled with mTORC2 pathway activation^45^. mTORC2 is a multiprotein complex of the mammalian serine/threonine kinase mammalian target of rapamycin (mTOR), which comprises mTOR, mLST8, mSin1 and Rictor. mTORC2 is the prime Serine 473 kinase of Akt^46^. Aside its overactivation in cancer, mTorc2 and Akt phosphorylation play central roles in various models of differentiation including neurogenesis^47,48^. Moreover, the overexpression of Nox4 in NPSCs increased the production of ROS and the phosphorylation of Akt and promotes neurogenesis in the hippocampus following injury^49^. Earlier, we described that PARP3 inactivation causes impaired mTORC2 signaling in breast cancer^26^. We therefore examined whether and how Parp3 deficiency and increased Nox4-generated ROS modulate mTorc2 activation in NPSCs and during astrocyte differentiation (Fig. 5a, b). While *Parp3*^*+/+*^ cultures displayed a remarkable and rapid increase in p-AktS473 throughout astrocytes differentiation, this increase was significantly compromised in *Parp3*^*–/*^^–^ cultures. We also analyzed the expression of Rictor, involved in mTorc2 stability and the phosphorylation status of Gsk3β on Ser9, a target of activated Akt^46^. The levels of both signals were significantly reduced in the *Parp3*^*–/*^^–^ NPSCs and astrocytes compared to the *Parp3*^*+/+*^ controls. We also detected less autophosphorylation of mTorc2 on Ser 2481 in *Parp3*^*–/*^^–^ astrocytes d6 and d8 compared to *Parp3*^*+/+*^ controls. mTorc2 autophosphorylation on Ser 2481 is defined as a marker of intact mTorc2 activity^46^. In contrast, the expression levels of the two other core components mSin1 and mLST8 were not affected by the absence of *Parp3* (Supplementary Fig. 5). These results suggest that Parp3 plays a role in maintaining a basal level of Rictor expression and mTorc2 activity in NPSCs and is required for efficient mTorc2 activation and Akt phosphorylation during differentiation to astrocytes.

Next, to demonstrate the contribution of Nox4-produced ROS in mTorc2 activation and astrocyte differentiation, we analyzed the status of p-AktS473 and p-Gsk3βS9, respectively, upon Nox4 depletion in NPSCs and astrocytes (Fig. 5c, d). The silencing of Nox4 had no impact on *Parp3*^*+/+*^ or *Parp3*^*–/*^^–^ NPSCs, nor *Parp3*^*+/+*^ astrocytes but restored p-AktS473 and p-Gsk3bS9 in *Parp3*^*–/*^^–^ astrocytes revealing that Nox4 mediates the contribution of Parp3 in mTorc2 activation during astrocyte differentiation.

### Enhanced Nox4-induced ROS in Parp3-deficient cells cause increased oxidation of Rictor provoking its Fbxw7-mediated ubiquitination and degradation

**Fig. 6:**
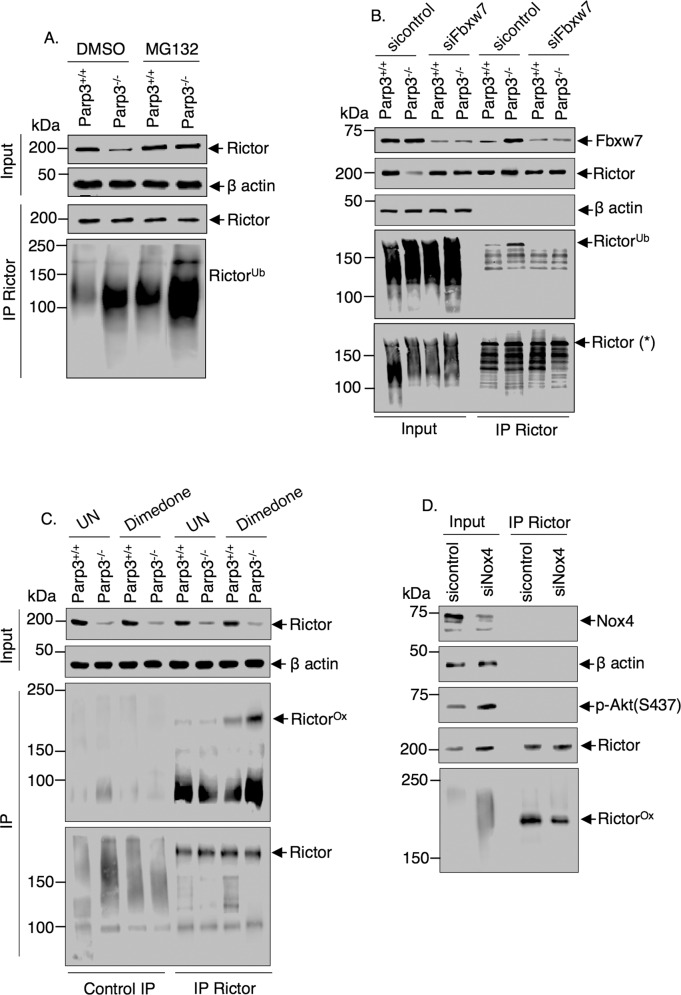
Enhanced Nox4-induced oxidative stress in *Parp3*^*–/*^^–^ NPSC causes increased oxidation of Rictor provoking its Fbxw7-mediated ubiquitination and degradation. **a** In vivo ubiquitination assay: The *Parp3*^*+/+*^ and *Parp3*^*–/*^^–^ NPSC were transfected with HA-Ubiquitin and mock-treated or treated with MG132 to inhibit proteasomal degradation. Rictor immunoprecipitates were blotted successively with an anti-HA antibody to detect ubiquitinated Rictor and a Rictor antibody to detect immunoprecipitated Rictor. **b** Enhanced co-immunoprecipitation of Fbxw7 with ubiquitinated Rictor in the *Parp3*^*–/*^^–^ compared to the *Parp3*^*+/+*^ NPSCs. Co-immunoprecipitation is significantly reduced in the Fbxw7-silenced cells because of the absence of ubiquitinated Rictor. *Parp3*^*+/+*^ and *Parp3*^*–/*^^–^ NPSC were transfected with either sicontrol or siFbxw7 and processed for in vivo ubiquitination as in **a**. **c** Increased cysteine-oxidized Rictor in *Parp3*^*-/-*^ NPSC compared to *Parp3*^*+/+*^ NPSC. *Parp3*^*+/+*^ and *Parp3*^*–/*^^–^ NPSC were left untreated (UN) or treated with a dimedone-based reagent to trap cysteine sulfenic acids resulting from oxidized cysteines. Immunoprecipitated Rictor were analyzed by immunoblotting using successively an anti-cysteine sulfenic acid antibody and an anti-Rictor antibody. Control immunoprecipitation was performed using an unrelated IgG antibody. **d** The silencing of Nox4 reduces the levels of cysteine-oxidized Rictor in *Parp3*^*–/*^^–^ NPSC. *Parp3*^*–/*^^–^ NPSC were treated with the dimedone-based reagent to trap cysteine sulfenic acids resulting from oxidized cysteines. Immunoprecipitated Rictor were analyzed by immunoblotting using an anti-cysteine sulfenic acid antibody (for Rictor^Ox^) and an anti-Rictor antibody. Inputs were analyzed for the levels of Nox4 to validate its depletion, p-Akt(S473) confirming increased Akt phosphorylation in siNox4-treated cells, and β-actin as a loading control.

In cancer cells, the levels of Rictor are modulated by an ubiquitination/prteasome-mediated degradation catalyzed by the E3 ubiquitin ligase FBXW7^50^. Therefore, to further unveil the biochemical basis of Rictor stability in our model, we analyzed the levels of ubiquitinated Rictor immunoprecipitates in the *Parp3*^*+/+*^ versus the *Parp3*^*–/*^^–^ NPSCs (Fig. 6a). The absence of Parp3 induced an apparent increase in the levels of ubiquitinated Rictor. To verify the contribution of Fbxw7, we studied the association of Fbxw7 with ubiquitinated Rictor and measured the levels of ubiquitinated Rictor upon silencing of Fbxw7 in the *Parp3*^*+/+*^ and *Parp3*^*–/*^^–^ NPSCs (Fig. 6b). In the experimental conditions where we favored ubiquitination, we detected an increased coimmunoprecipitation of Fbxw7 with HA-Ub-Rictor in the *Parp3*^*–/*^^–^ cells compared to the *Parp3*^*+/+*^ cells. This association was lost upon Fbxw7 depletion because of the reduced levels of ubiquitinated Rictor. We also revealed enhanced coimmunoprecipitation of Fbxw7 with Rictor in basal conditions in the absence of Parp3 which was reduced in Fbxw7-silenced cells. These results confirm that in the absence of Parp3, Rictor is guided to Fbxw7-mediated ubiquitination and proteasomal degradation that weakens the mTorc2 complex and activity. Knowing that oxidation-induced conformational changes often drive ubiquitination and clearance of redox-sensitive proteins^51,52^, we hypothesized that the enhanced Nox4-induced mitochondrial stress may lead to the oxidation of Rictor and consequently its ubiquitination-mediated degradation. To assess the oxidation of Rictor, we in situ labeled the oxidized cysteine residues using dimedone, which reacts with the thiol groups of cysteines (Fig. 6c). Immunoprecipitation of Rictor revealed higher levels of oxidized Rictor in the *Parp3*^*–/*^^–^ versus the *Parp3*^*+/+*^ NPSCs. To verify the contribution of Nox4, we analyzed the levels of immunoprecipitated oxidized Rictor in dimedone-treated *Parp3*^*–/*^^–^ cells upon Nox4 silencing (Fig. 6d). The depletion of Nox4 significantly decreased the levels of oxidized Rictor compared to the sicontrol-treated cells. Together, these results support the hypothesis that the accumulation of toxic levels of ROS caused by the upregulation of Nox4 in *Parp3*^*–/*^^–^ cells contributes to the decreased activity of mTorc2 because of an oxidation-induced and ubiquitination-mediated degradation of Rictor.

### Parp3 deficiency causes reduced mTorc2 activity specifically in the striatum of naïve mice and after cerebral hypoxia-ischemia (HI)

**Fig. 7:**
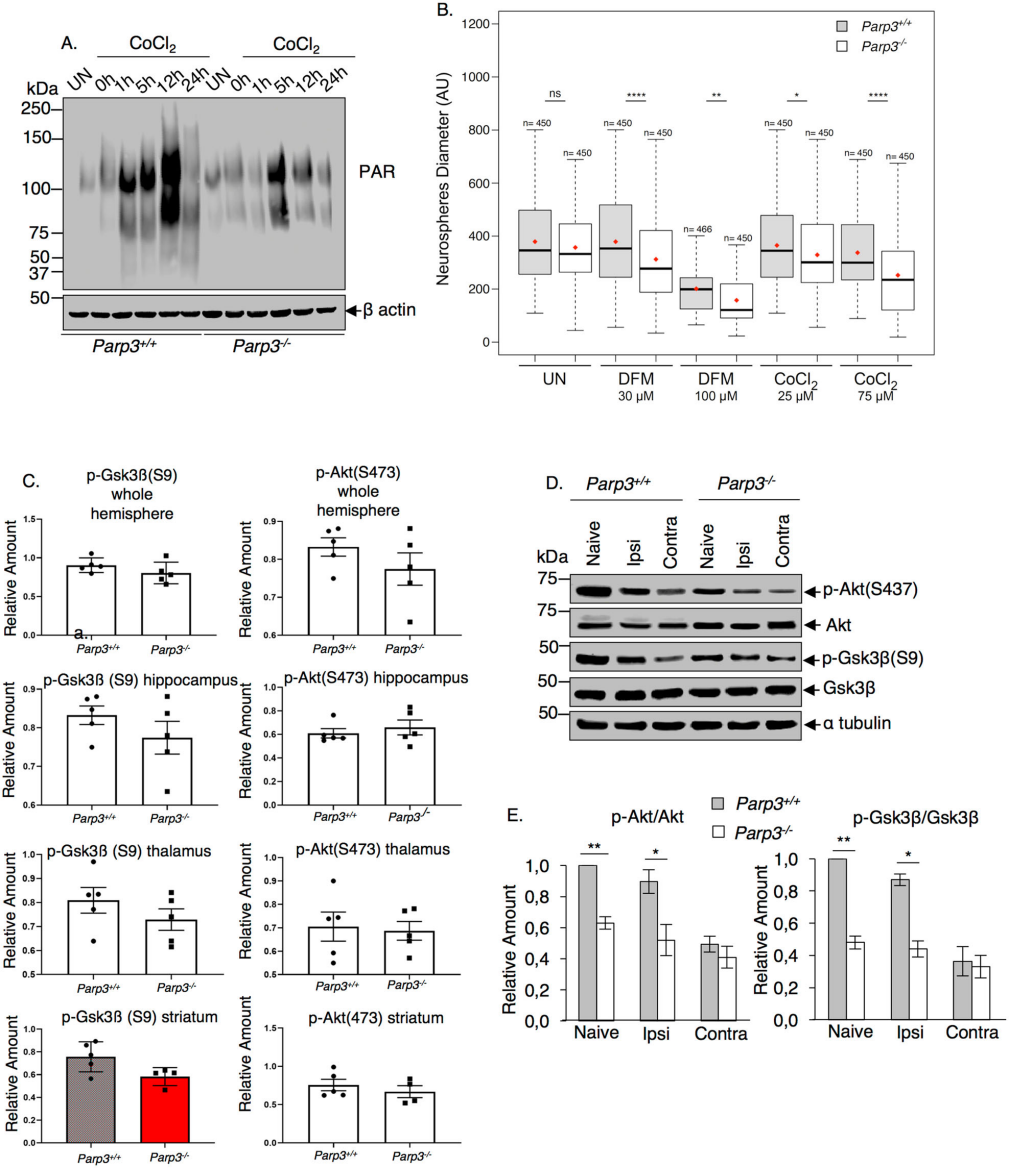
Reduced mTorc2 signaling in the striatum of post-natal Parp3-deficient mice and shortly upon hypoxia-ischemia. **a**, **b** Parp3 is involved in cell response to hypoxia. **a** Western blot analysis of ADP-ribose formation in *Parp3*^*+/+*^ and *Parp3*^*–/*^^–^ NPSCs at the indicated time points upon exposure to CoCl_2_ and in the untreated (UN) cells. **b** Diameter of neurospheres formed in *Parp3*^*+/+*^ and *Parp3*^*–/*^^–^ cultures 7 days upon exposure to the hypoxia mimetics CoCl_2_ and DFM for 48 h compared to untreated cultures (UN). Means are indicated in the boxplots as the red dots. Countings are from three independent experiments and three independent clones. *P < 0.05, **P < 0.01, ****P < 0.0001. **c** Immunostaining with anti p-Gsk3β (S9) and anti p-Akt (S473) of brain regions on ipsi and contralateral side of the ischemic event 6 h after cerebral hypoxia ischemia. Staining intensity was measured as a correlate of immunohistochemical reactivity in the ipsilateral relative to the contralateral side. Data are mean values ± s.d from 5 Parp3^+/+^ and 6 Parp3^*–/*^^–^ mice. **d** Western blot analysis for p-Akt (S473) versus Akt and p-Gsk3β (S9) versus Gsk3β and α-tubulin as loading control in total protein extracts of striatal biopsies from *Parp3*^*+/+*^ and *Parp3*^*–/*^^–^ mice either untreated (naïve) or 6 h upon exposure to HI. **e** Bar graphs depict the relative signal intensities of p-Akt versus Akt, p-Gsk3β versus Gsk3β, measured in three independent experiments and three mice of each genotype using Image J. Mean values ± s.d. are indicated. *P < 0.05, **P < 0.01.

Nox4-induced oxidative stress is a pathological feature of cerebral hypoxia-ischemia^53,54^ and Rictor/mTorc2 deficiency has been shown to aggravate HI-induced injury in various models^55,56^. Based on this knowledge, we aimed to verify the involvement of the Parp3-mTorc2 axis in vivo in normal mice and in response to HI. We first analyzed the expression pattern of ADP-ribose synthesis upon exposure of NPSCs to the hypoxia-mimetic agent Cobalt Chloride (Fig. 7a and Supplementary Fig. 7). We detected a gradual upregulation in poly(ADP-ribose) formation in the *Parp3*^*+/+*^ NPSCs that was temporarily reduced in the *Parp3*^*–/*^^–^ NPSCs implying a time-controlled contribution of Parp3-induced ADP-ribose synthesis in response to chemically induced hypoxia. We then examined the sensitivity of the *Parp3*^*+/+*^ and *Parp3*^*–/*^^–^ NPSCs to CoCl_2_ and desferrioxamine (DFM)-induced hypoxia (Fig. 7b and Supplementary Fig. 6). The absence of Parp3 rendered NPSCs hypersensitive to both drugs most significantly at the highest doses reducing their proliferation and survival, and uncovering an important role of PARP3 in response to hypoxia.

Next, to explore the Parp3-mTorc2 pathway in vivo, we analyzed mTorc2 activity in specific brain subregions of *Parp3*^*+/+*^ and *Parp3*^*–/*^^–^ mice 6 h after cerebral HI (Fig. 7c–e). We applied the widely used Levine method modified for use in perinatal mice^57^. A combination of hypoxia and cerebral ischemia produces injury confined to the brain hemisphere ipsilateral to the occluded common carotid artery. Immunostaining did not show differences in the levels of p-Akt(S473) of wild type and mutant mice in the different brain regions analysed (e.g., hippocampus, striatum and thalamus) but revealed a significantly reduced p-Gsk3β(S9) staining specifically in the striatum of the *Parp3*^*–/*^^–^ mice compared to the *Parp3*^*+/+*^ mice. No significant difference of p-Gsk3β(S9) staining were detected in the other sub-regions (Fig. 7c). Further, western blot analysis of these markers in protein extracts from striatal biopsies showed reduced p-Gsk3β(S9) and p-Akt(S473) in the striatum of the naïve animals and after cerebral HI (Fig. 7d, e). In sum, these results confirmed a prominent role of Parp3-regulated mTorc2 signaling in vivo in striatum of naïve animals and after post-natal HI.

## Discussion

Neurogenesis in the adult brain throughout life involves a tightly regulated balance between reactive oxygen species (ROS) generation and scavenging^58^. Numerous studies have shown that under normal physiological conditions, ROS act as indispensable regulators of intracellular signaling to promote NPSC proliferation, differentiation and hence brain development and function^1,2^. Emerging evidence suggest that astrocytic mitochondrial ROS are important for brain metabolism and neuronal function^9^. In contrast, oxidative stress that results from elevated levels of ROS has been established as a key contributor of neurodegenerative diseases including Parkinson, Alzheimer or ageing, or cell death in ischemia-reperfusion injury. Moreover, the principal regulator of ROS production in the central nervous system are the NOX family of NADPH oxidases^59^. Still the understanding of the molecular events that control their activity and hence the redox homeostasis are unclear.

In this study, we demonstrate that the Poly(ADP-ribose) polymerase Parp3 controls astrocytic differentiation via NF-kB regulated Nox4-induced ROS production. Moreover, the process implicates a specific regulation of Nox4-dependent activation of mTorc2, a crosstalk previously identified in pulmonary artery smooth muscle cells^60^.

We show that the absence of Parp3 results in high pathological levels of Nox4-generated ROS that compromise efficient astroglial differentiation of NPSCs. We have not yet identified how Parp3-deficiency mediates increased nuclear translocation of NF-kB and Nox4 induction. However, taking into account that accumulation of DNA damage promotes NF-kB activation^61,62^, we speculate that Parp3-deficient NPSC and astrocytes display enhanced genome instability. Consistently, we detected higher levels of γH2AX expression in *Parp3*^*–/*^^–^ NPSC and astrocytes (Supplementary Fig. 8). In line with this, it has been demonstrated that PARP3 is involved in the activation of ATM, a key regulator of genotoxic stress-induced NF-kB signaling^63^. The upregulation of Nox4 has previously been associated with mitochondrial dysfunction in cardiac myocytes^39,64^. Similarly, we detected increased mitochondrial production of ROS and an impaired mitochondrial membrane potential in the Parp3-deficient astrocytes indicating mitochondrial dysfunction in our model. Mitochondrial ROS overload is restored to normal upon Nox4 silencing implying the causative role of enhanced Nox4 activity in Parp3-deficient astrocytes. Importantly, mitochondrially-derived oxidative stress has been implicated in the oxidation of Rictor resulting in the inactivation of mTorc2 and impaired Akt(S473) phosphorylation^65^. Consistently, our findings reveal that increased Nox4-generated ROS leads to dysfunctional mTorc2 signaling including attenuation of Akt(S473) phosphorylation during astrocytic differentiation of *Parp3*^*–/*^^–^ NPSCs. More specifically, we identified increased oxidation and Fbxw7-mediated ubiquitination and degradation of Rictor in the *Parp3*^*–/*^^–^ NPSCs. Moreover, in line with the role of oxidation-induced conformational changes in the ubiquitination and clearance of redox-sensitive proteins^51,52^, our findings demonstrate the contribution of Nox4-induced oxidative stress in the oxidation of Rictor and consequently its ubiquitination-mediated degradation resulting in a decrease in mTorc2 activity.

The analysis of the transcriptomic data revealed that genes coding for proteins involved in the ECM structure and development including proteoglycans and glycoproteins are predominantly affected in the Parp3-deficient astrocytes. It has been extensively reported that ECM molecules produced by astrocytes play instrumental roles in the establishment and function of synapses and in neuronal activity and plasticity during development and in the mature brain, or in brain regeneration upon injury^66–69^. Consistently, several of the Parp3-regulated transcripts are associated with synapse activity. It is also established that increased levels of ROS can alter ECM properties at multiple levels. A focus on degenerative chronic diseases highlighted the specific role of NADPH oxidase-mediated ROS production in ECM degradation^70,71^. Based on these observations, our findings suggest that Parp3 controls the production and function of ECM by regulating the overdose of Nox4-generated ROS which has fundamental implications in the astroglial differentiation and activation.

In vivo, we find that the alteration of mTorc2 signaling prevails in the striatum of the naïve post-natal Parp3-deficient mice or shortly after hypoxia-ischemia. Moreover, increased ROS, reduced p-Akt(S473) signaling and altered astrocytic differentiation is confirmed in the *Parp3*^*–/*^^–^ NPSC isolated from the subventricular zone, an important germinal zone for striatal neurogenesis (Supplementary Fig. 9). These findings imply that Parp3 confers a brain-region-specific regulation of the ROS balance and the mTorc2 complex and indicate a higher vulnerability in the striatum of the *Parp3*-deficient mice. Because an overload of ROS production is associated with a variety of CNS diseases throughout aging, we speculate that the deleterious effects of reduced mTorc2 activity might become more pronounced in aged animals. Moreover, given the altered mTorc2 signaling after cerebral hypoxia-ischemia detected here and the role of oxidative stress to stroke injury, we may speculate that *Parp3*^*–/*^^–^ mice are more vulnerable to HI.

In sum, our work unveils an important role of Parp3 in promoting efficient astroglial differentiation that implicates a fine-tuned regulation of striatal Nox4-derived ROS and mTorc2 activation. Whether this role of Parp3 has implications in CNS development or related behavior or in the regeneration of the striatum after HI merits further investigations. If so, the relevance of PARP3 inhibition in clinical therapeutic trials for cancer treatment has to been taken with care.

## Supplementary information

Supplementary material

Supplementary Table 1

Supplementary Table 2

Supplementary Figure 1

Supplementary Figure 2

Supplementary Figure 3

Supplementary Figure 4

Supplementary Figure 5

Supplementary Figure 6

Supplementary Figure 7

Supplementary Figure 8

Supplementary Figure 9

Supplementary figure legends
