# Supplementary material for: Parp3 promotes astrocytic differentiation through a tight regulation of Nox4-induced ROS and mTorc2 activation
Source: Cell Death Dis. 2020 Nov 6;11(11):954. doi: 10.1038/s41419-020-03167-5 (PMC7648797; doi:10.1038/s41419-020-03167-5)
Supplement: Supplementary file 1 — Supplementary material [file 41419_2020_3167_MOESM1_ESM.docx]

**Supplementary material and methods**

**Ethical approvement for animal use**

Animal experiments have been approved by FOTS (Forsøksdyrforvaltningens tilsyns- og søknadssystem) as the experimental animal administration's supervision and application system of Norway. Project ID 7261.

**Neurosphere cultures and treatments**

For self-renewal assays and neurosphere size determination (related to Supplementary Fig.1), single-cell suspensions were replated at 40 cells.μl^-1^ in 6 well plates. The number and size of primary, secondary, tertiary or quaternary spheres were determined after 7 days *in vitro* culture. For each genotype, the mean neurospheres-forming frequency was obtained from three biological replicates and three independent clones by counting at least 10 independent fields by replicate and cell group. The mean neurospheres diameter (Arbitrary Unit, AU) is obtained from 150 randomly chosen neurospheres by replicate per clone and analysed using Image-J software.

For treatment with the chemical agents (related to Supplementary Fig. 3 and 5), viable single cells were seeded at 1,5.10^6^ cells.ml^-1^ 24h before treatment. Cells were treated with 10 μM paraquat (PQ, Sigma), 1 μM menadione (Menad. Sigma) or 1 μM H_2_O_2_ (Sigma) for 1h and with CoCl_2_ (Sigma) or Deferroxamine Mesylate Salt (DFM, Sigma) at the indicated concentrations for 24h. Medium was renewed and cells were processed as indicated.

**Calcein AM viability assay**

(Related to Supplementary. Fig. 2). Single NSPC cells were seeded at 4x10^4^ cells per well in 24- black-walled plates and processed for differentiation to astrocytes. At the indicated time points, cells were processed for calcein-AM staining (Interchim Flow probes) and calcein fluorescence production was measured using the Typhoon FLA 9500 Biomolecular Imager.

**Acid extracts**

For the analysis of γH2AX and H2AX (related to Supplementary Fig. 6), exponentially growing cells were collected and lysed by incubation on ice for 10 minutes in Triton-Extraction Buffer (TEB) (PBS containing 0.5% Triton X100, 2mM PMSF, 0.02% NaN3) at a density of 1.10^7^ cells/mL. After centrifugation at 6500g at 4°C for 10 minutes, nuclei pellets were washed once in TEB, resuspended in 0.2 N HCl at a density of 4.10^7^ nuclei/mL and acid extracted overnight at 4°C. After centrifugation at 6500g at 4°C for 10 minutes, cleared suspension were treated with 2M NaOH at 1/10 the volume of the suspension and protein content was quantified by Bradford protein assay. Proteins were analysed by 10% SDS-PAGE and immunoblotting using the appropriate antibodies.

**Preparation of total RNA libraries and sequencing**

Cytoplasmic and mitochondrial ribosomal RNA (rRNA) was removed using biotinylated, target-specific oligos combined with Ribo-Zero rRNA removal beads. Following purification, the depleted RNA was fragmented into small pieces using divalent cations at 94°C for 2 minutes. Cleaved RNA fragments were then copied into first strand cDNA using reverse transcriptase and random primers followed by second strand cDNA synthesis using DNA Polymerase I and RNase H. Strand specificity was achieved by replacing dTTP with dUTP during second strand synthesis. The double stranded cDNA fragments were blunted using T4 DNA polymerase, Klenow DNA polymerase and T4 PNK. A single 'A' nucleotide was added to the 3' ends of the blunt DNA fragments using a Klenow fragment (3' to 5'exo minus) enzyme. The cDNA fragments were ligated to double stranded adapters using T4 DNA Ligase. The ligated products were enriched by PCR amplification (30 sec at 98°C; [10 sec at 98°C, 30 sec at 60°C, 30 sec at 72°C] x 12 cycles; 5 min at 72°C). Surplus PCR primers were further removed by purification using AMPure XP beads (Beckman-Coulter, Villepinte, France) and the final cDNA libraries were checked for quality and quantified using capillary electrophoresis.

For sequencing, Cutadapt 1.10 (-- adapter AGATCGGAAGAGCACACGTCTGAACTCAGTCAC--quality-cutoff 20,20--anywhere"A(100)--minimum-length 40) was used for read preprocessing: adapter, poly-A and low quality (Phred quality score below 20) bases trimming, removal of readers shorter than 40 bp after trimming. Reads mapping to spike and rDNA sequences were also discarded. Reads were mapped onto the mm10 assembly of Mus musculus genome using STAR version 2.5.3a (--twopassMode Basic) (28). Gene expression was quantified using htseq-count release 0.6.1p1 (29)(--mode union –minaqual 10) and gene annotations from Ensembl release 92.
