# Supplementary material for: Parp3 promotes astrocytic differentiation through a tight regulation of Nox4-induced ROS and mTorc2 activation
Source: Cell Death Dis. 2020 Nov 6;11(11):954. doi: 10.1038/s41419-020-03167-5 (PMC7648797; doi:10.1038/s41419-020-03167-5)
Supplement: Supplementary file 2 — Supplementary Table 1 [file 41419_2020_3167_MOESM2_ESM.docx]

**Supplementary Table 1:** List of antibodies used in the study

| **Name** | **Company + number** | **Concentration** |
| --- | --- | --- |
| Rabbit anti-PARP3 (4698) | Home made | WB 1:10000 |
| Mouse anti-PARP1 (EGT69) | Home made | WB 1:10000 |
| Rabbit anti-Poly(ADP-ribose) | Trevigen  4336-BPC-100 | WB 1:1000 |
| Mouse anti-GFAP (GA5) | Millipore MAB360 | WB 1:1000 |
| Mouse anti-SOX2 | R&D Systems MAB2018 | WB 1:1000 |
| Rabbit anti-NANOG | Thermo Fisher Scientific  PA1-097 | WB 1:1000 |
| Rabbit anti-phospho mTORC2 (S2481) | Cell Signaling #2974 | WB 1:1000 |
| Rabbit anti-mTOR | Cell Signaling #2972 | WB 1:1000 |
| Mouse anti-Rictor (H-11) | Santa Cruz Biotech  sc-271081 | WB 1:1000, IP 1:100 |
| Rabbit anti-Rictor | Sigma SAB4200141 | WB 1:2000 |
| Rabbit anti-cystein sulfenic acid | Millipore 07-2139 | WB 1:10000 |
| Mouse anti-phospho Akt (S473) | Santa Cruz Biotech  Sc-293125 | WB 1:1000 |
| Goat anti-Akt (C-20) | Santa Cruz Biotech sc-1618 | WB 1:1000 |
| Rabbit anti-phospho GSκ3β (S9) (53B) | Cell Signaling #9323 | WB 1:1000 |
| Rabbit anti-GSκ3β (27C20) | Cell Signaling #9315 | WB 1:1000 |
| Rabbit anti-HIF1α | Bethyl Labs A300-286A | WB 1:1000 |
| Mouse anti-HA (6E2) | Cell Signaling #2367 | WB 1:1000 |
| Rabbit anti-FBXW7 (MR) | Aviva Systems Biology ARP37443_P050 | WB 1:750 |
| Rabbit anti-βactin | Sigma-Aldrich A2066 | WB 1:10000 |
| Mouse anti-αtubulin | Sigma Aldrich T9026 | WB 1:10000 |
| Rabbit Lamin B1, B-5-1-2 | Abcam ab16048 | WB 1:1000 |
| Rabbit NOX4 | GeneTex International GTX121929 | WB 1:1000 |
| Rabbit DUOX1 | Abbexa Technology abx124235 | WB 1:1000 |
| Mouse anti-phospho Akt (S473) (C11) | Santa Cruz Biotech  Sc-514032 | IHC 1:50 |
| Rabbit anti-Akt | Sigma Aldrich SAB4500798 | IHC 1:100 |
| Rabbit anti-phospho GSκ3β (S9) (5B3) | Cell Signaling #9323 | IHC 1:50 |
| Rabbit anti-GSκ3β (3D10) | Cell Signaling #9315 | IHC 1:50 |
| Mouse anti-NF-kB p65 | Santa Cruz Biotech sc8008 | WB 1:1000 |
| Rabbit anti-Sin1 | Cell Signaling #12860 | WB 1:1000 |
| Rabbit anti-LST8 | Cell Signaling #3227 | WB 1:1000 |
