# Supplementary material for: Parp3 promotes astrocytic differentiation through a tight regulation of Nox4-induced ROS and mTorc2 activation
Source: Cell Death Dis. 2020 Nov 6;11(11):954. doi: 10.1038/s41419-020-03167-5 (PMC7648797; doi:10.1038/s41419-020-03167-5)
Supplement: Supplementary file 3 — Supplementary Table 2 [file 41419_2020_3167_MOESM3_ESM.docx]

**Supplementary Table 2:** List of RT-qPCR primers used in the study

| Gene | Sequences |
| --- | --- |
| *Parp3* | Fwd 5’-TGGCAAGGGCATCTACTTTG-3’  Rev 5’-TCCGTGTTGATATGGTGCTC-3’ |
| *Sox2* | Fwd 5’-CATGGGCTCTGTGGTCAAGT-3’  Rev 5’-CGGGGAGGTACATGCTGATC-3’ |
| *Oct4* | Fwd 5’-GAGAAGTGGGTGGAGGAAGC-3’  Rev 5’-CTCCACCTCACACGGTTCTC-3’ |
| *Nanog* | Fwd 5’-GAAGTACCTCAGCCTCCAGC-3’  Rev 5’-CACTGGTTTTTCTGCCACCG-3’ |
| *Gapdh* | Fwd 5’-AGCTTGTCATCAACGGGAAG-3’  Rev 5’-TTTGATGTTAGTGGGGTCTCG-3’ |
| *Gfap* | Fwd 5’-TCCTGGAACACGAAAACAAG-3’  Rev 5’-CAGCCTCAGGTTGGTTTCAT-3’ |
| *Nox4* | Fwd 5’-AAACACCTCTGCCTGCTCAT-3’  Rev 5’-CGCCCAACATTTGGTGAATG-3’ |
| *Duox1* | Fwd 5’-ACCAAGCCAACCTTTCCA-3’  Rev 5’-ACAGCCTCGTCTTCCCAC-3’ |
| *Cspg4* | Fwd 5′-AATGAGGACCTGCTACACGG-3′,  Rev 5′- CATCTGTAGTCAACAGCCGC-3′ |
| *Tnc* | Fwd 5’-ACCATGCTGAGATAGATGTTCCAAA-3’  Rev 5’- CTTGACAGCAGAAACACCAATCC-3’ |
| *SerpinH1* | Fwd 5’-CTGCAGTCCATCAACGAGTGGGC-3’  Rev 5’-ATGGCGACAGCCTTCTTCTGC-3’ |
| *Col11a1* | Fwd 5’-CTGGTCATCCTGGGAAAGAA-3’  Rev 5’TTGAATCCTGGAAAGCCATC-3’ |
| *Col5a1* | Fwd 5’-TGCCCTCTGACTGCCTCTAT-3’  Rev 5’-CACATTGCAGCCTGAAAGAA-3’ |
| *Col8a1* | Fwd 5’-AACTACAACCCGCAGACAGG-3’  Rev 5’-TGAATAGAGCAACCCACACG-3’ |
| *Ank3* | Fwd 5’-CTGGTAAAGAGACATAAACTGGC-3’  Rev 5’-CCATTGAGAAGCTCCGCGAG-3’ |
| *Adamts14* | Fwd 5’-AGCCTGGCCTACAAGTACGTCAT-3’  Rev 5’-CTCCTCCACAGGCCTTGCTGCA-3’ |
| *Adamts1* | Fwd 5’-CTGGCAGAAACAACACAACAG-3’  Rev 5’-TGAATTGGGCCATGTGTTTAAC-3’ |
| *Adamts20* | Fwd 5’-AATGTGCCAAGGTCTTCATAGA-3’  Rev 5’-AAACAGTGGCATCGGTAAGT-3’ |
| *Flot2* | Fwd 5’-AGGCTGTTGTGGTTCTGACTA-3’  Rev 5’-TGCAACGTCATAATCTCTAGGGA-3’ |
| *Grm4* | Fwd 5’-GGCCCTCAAGTGGAACTATG-3’  Rev 5’-CTCGTTGGCAAAGATGATGA-3’ |
| *Grin2a* | Fwd 5’-ATTCAACCAGAGGGGCGTA-3’  Rev 5’-TTCAAGACAGCTGCGTCATAG-3’ |
| *Shisa 6* | Fwd 5’-GCCAGCGCGACTGTTACTTAC-3’  Rev 5’-TCGCTGTTGTTGCACTCGA-3’ |
