# Supplementary figures and images for: Parp3 promotes astrocytic differentiation through a tight regulation of Nox4-induced ROS and mTorc2 activation
Source: Cell Death Dis. 2020 Nov 6;11(11):954. doi: 10.1038/s41419-020-03167-5 (PMC7648797; doi:10.1038/s41419-020-03167-5)

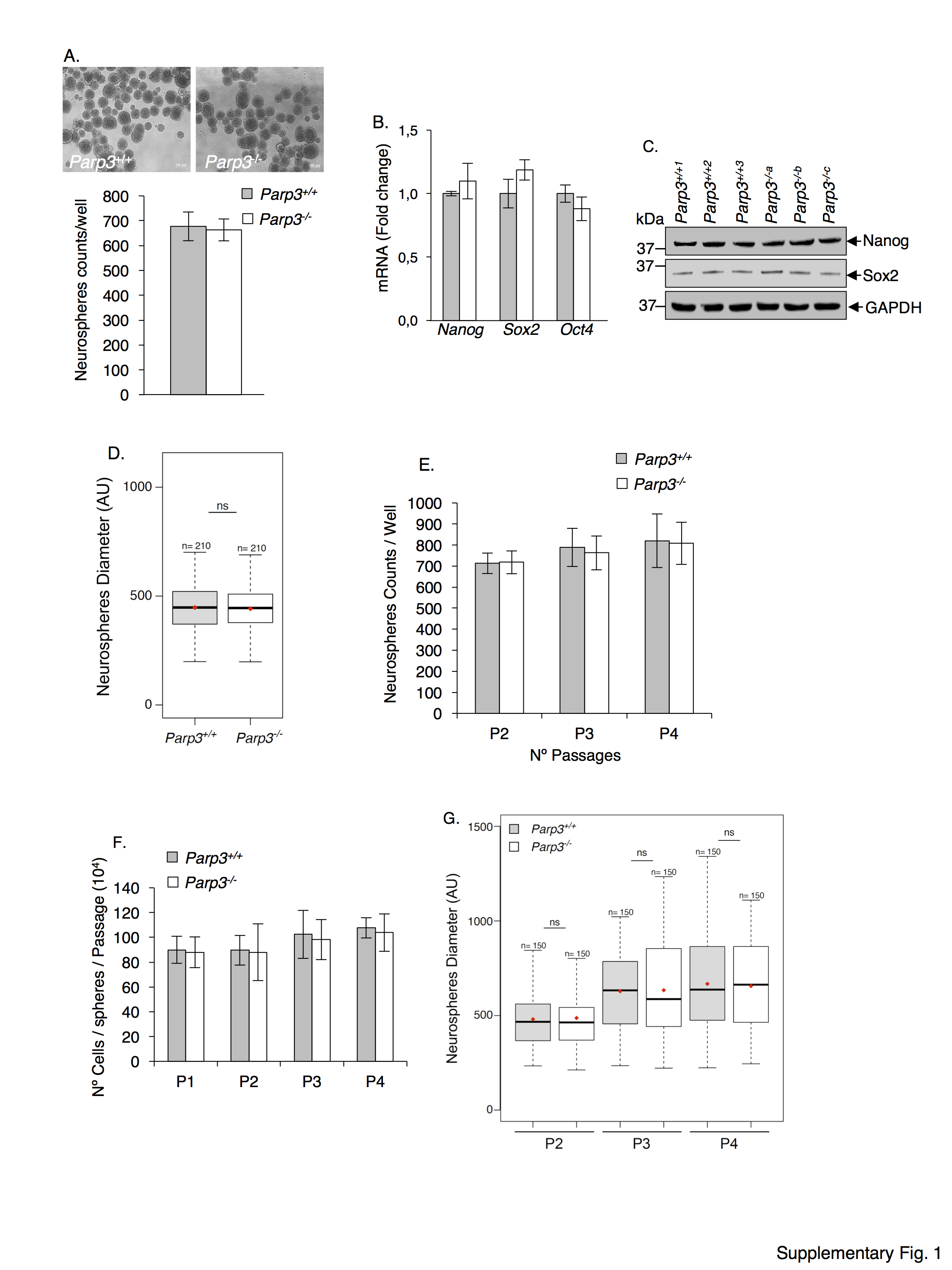

Supplement: Supplementary file 4 — Supplementary Figure 1 [file 41419_2020_3167_MOESM4_ESM.png]

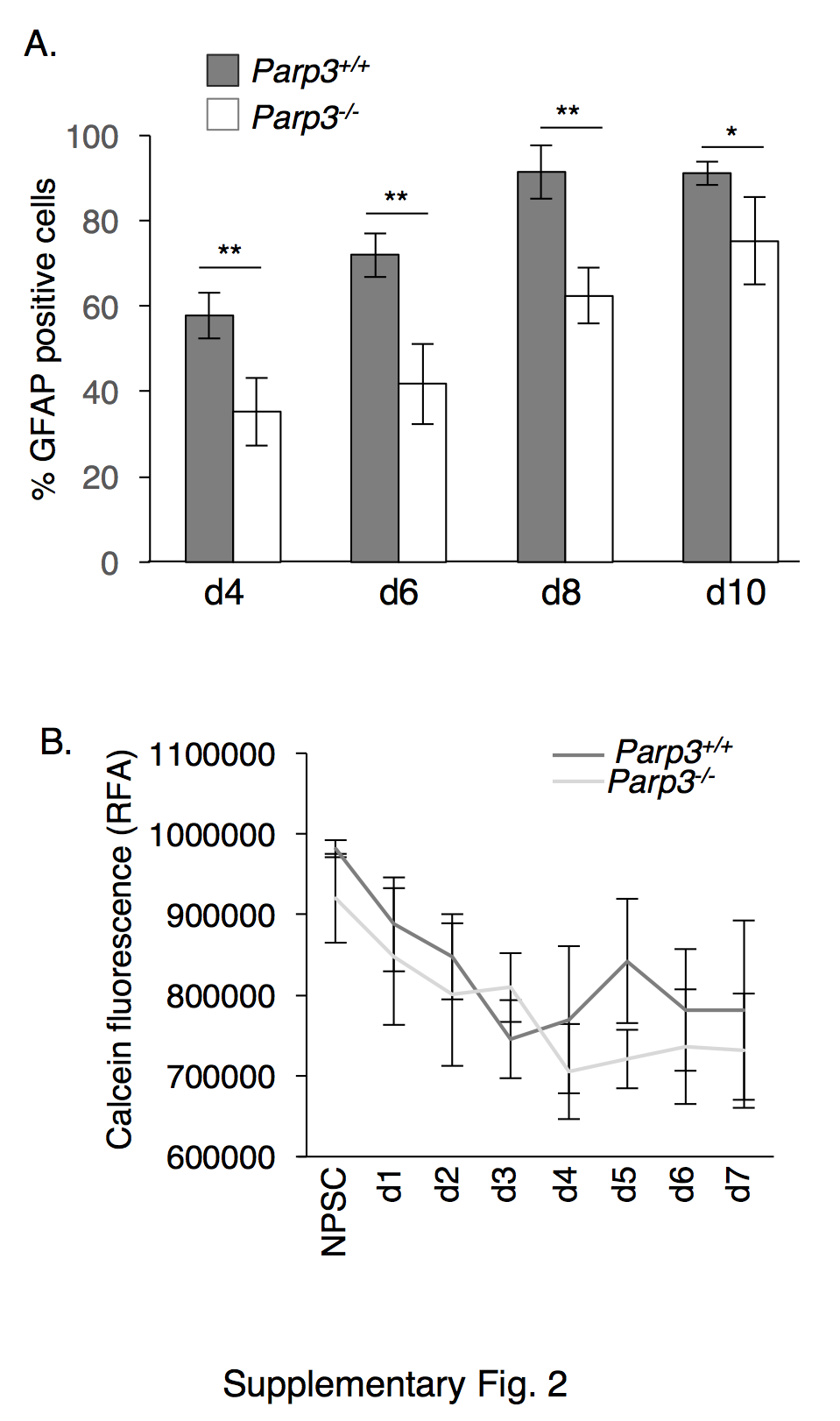

Supplement: Supplementary file 5 — Supplementary Figure 2 [file 41419_2020_3167_MOESM5_ESM.png]

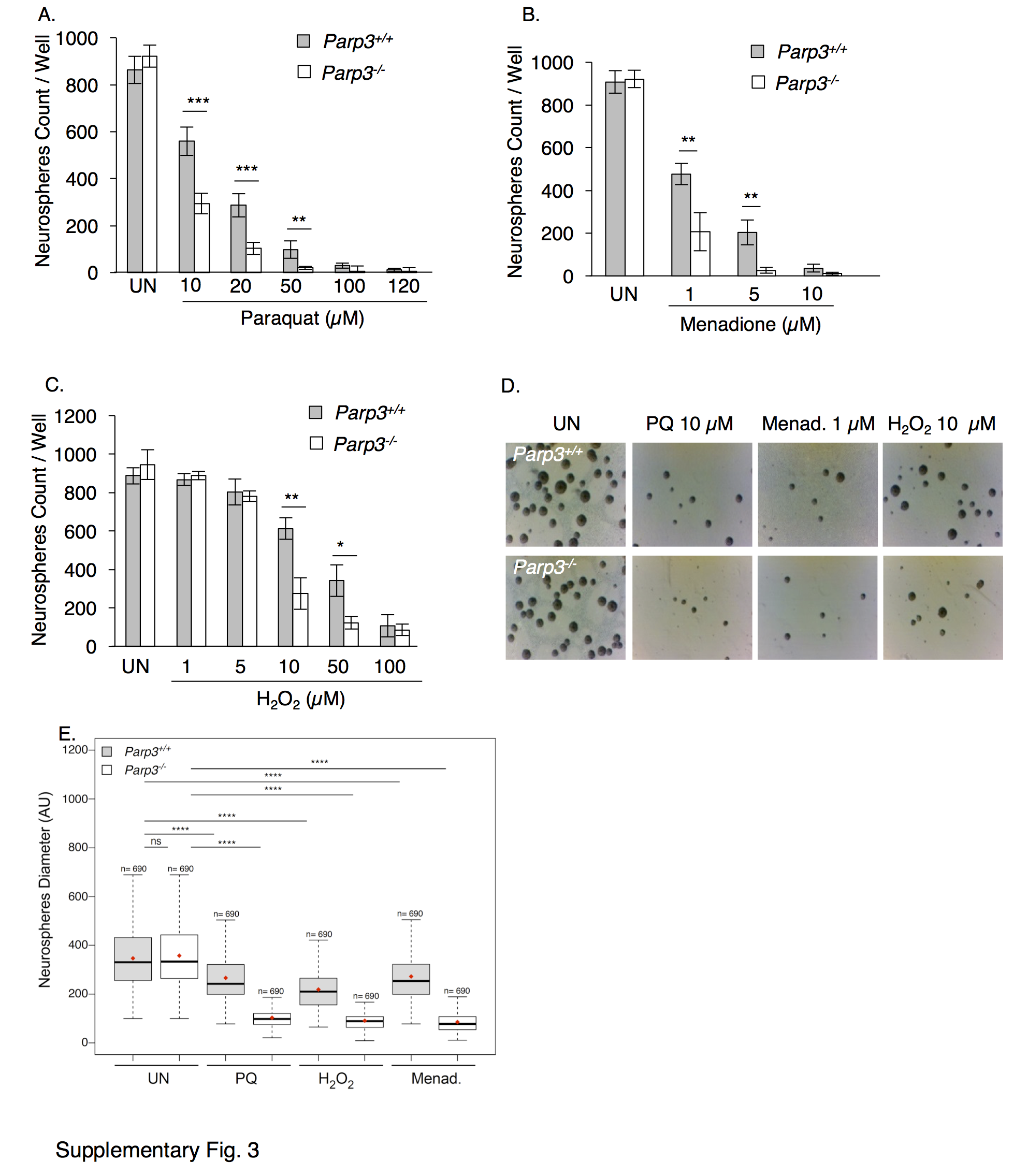

Supplement: Supplementary file 6 — Supplementary Figure 3 [file 41419_2020_3167_MOESM6_ESM.png]

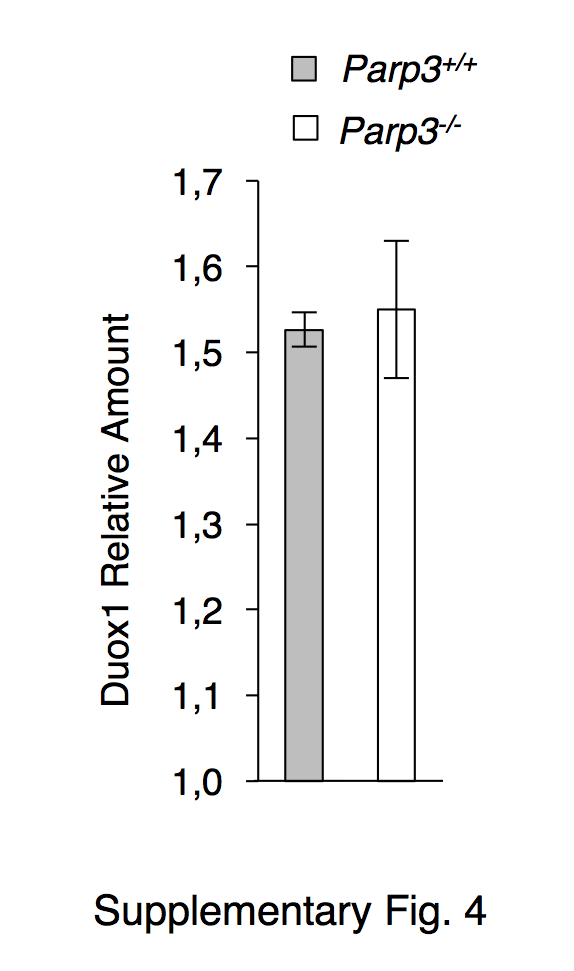

Supplement: Supplementary file 7 — Supplementary Figure 4 [file 41419_2020_3167_MOESM7_ESM.png]

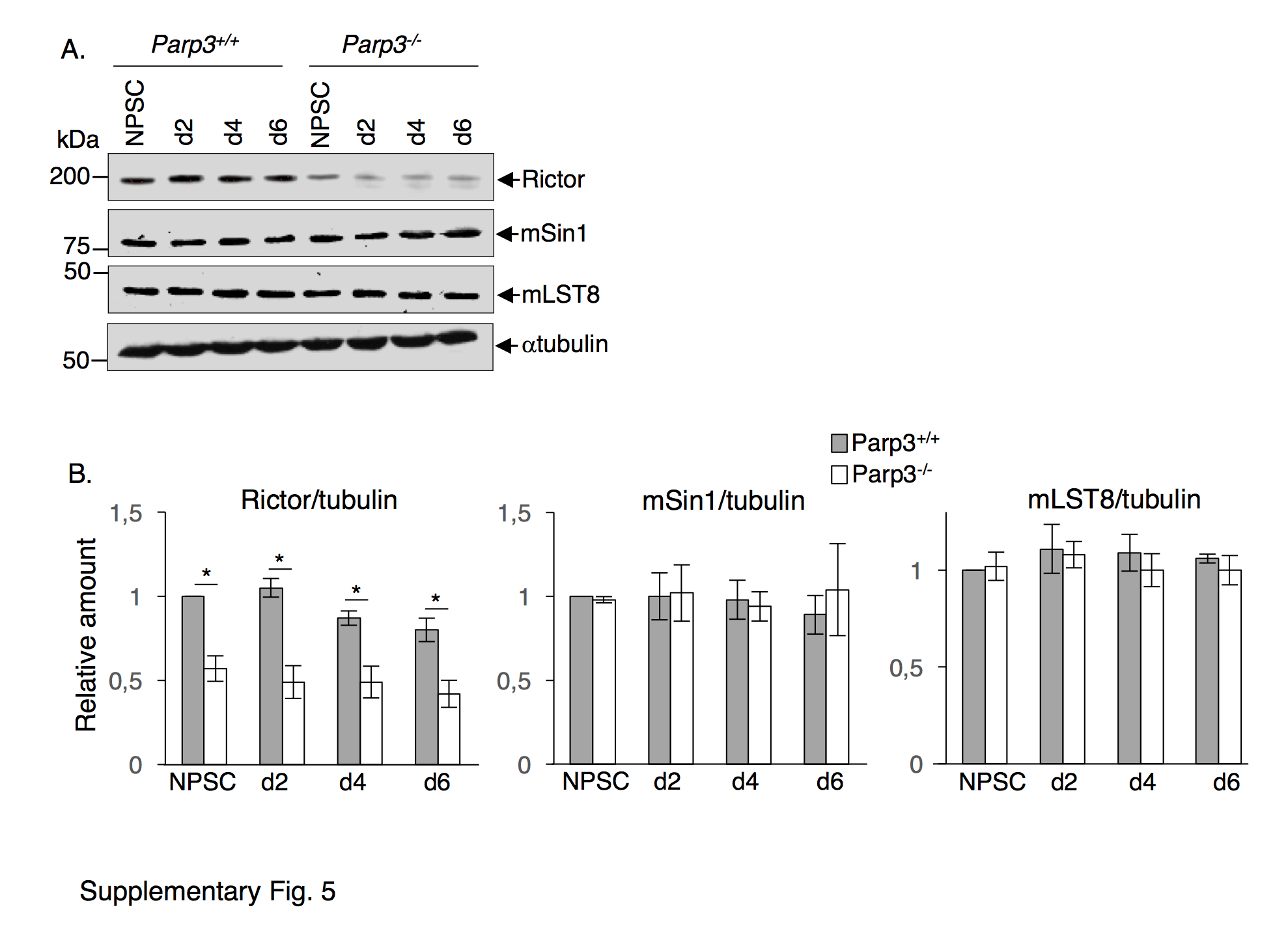

Supplement: Supplementary file 8 — Supplementary Figure 5 [file 41419_2020_3167_MOESM8_ESM.png]

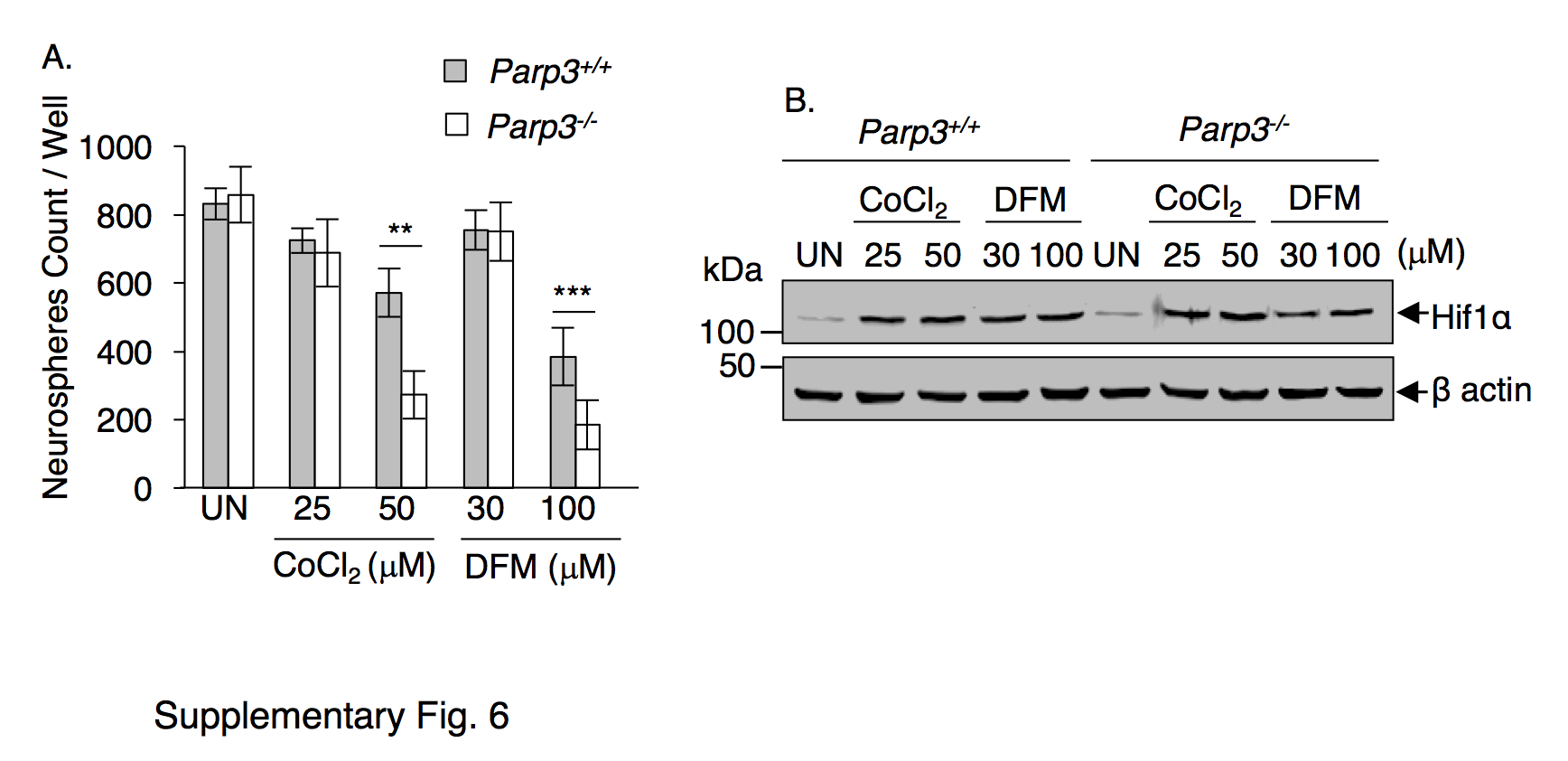

Supplement: Supplementary file 9 — Supplementary Figure 6 [file 41419_2020_3167_MOESM9_ESM.png]

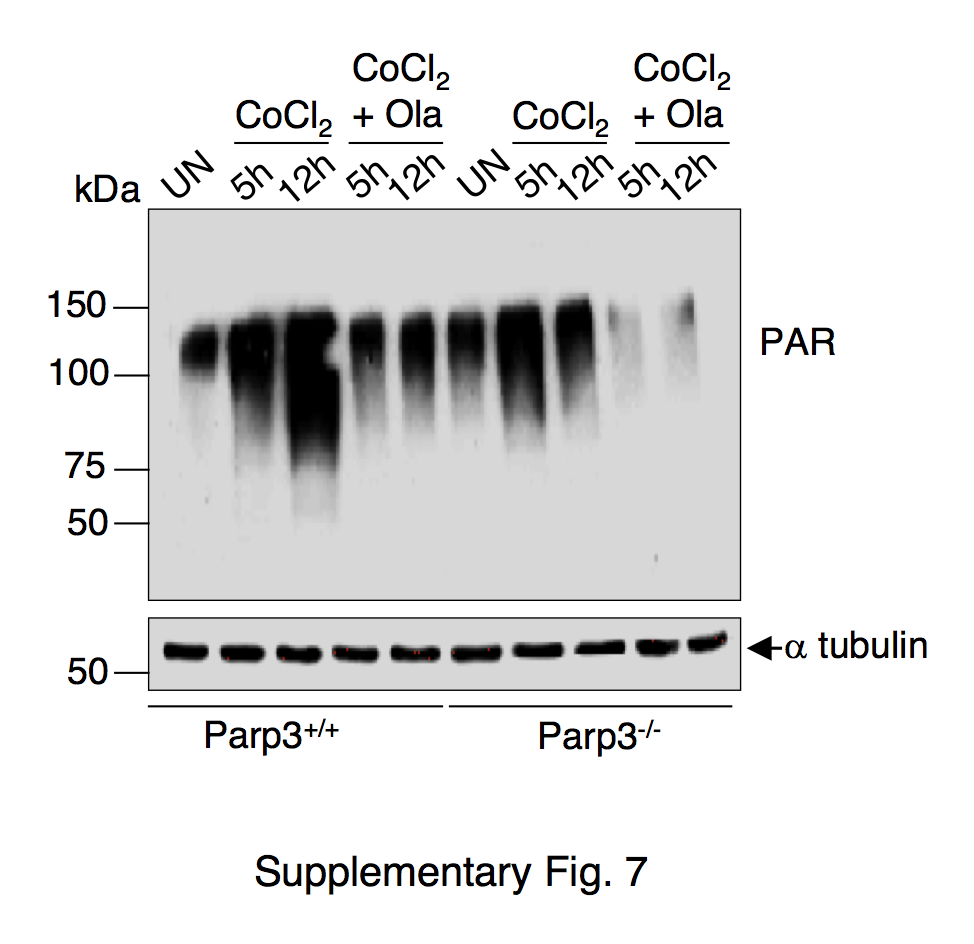

Supplement: Supplementary file 10 — Supplementary Figure 7 [file 41419_2020_3167_MOESM10_ESM.png]

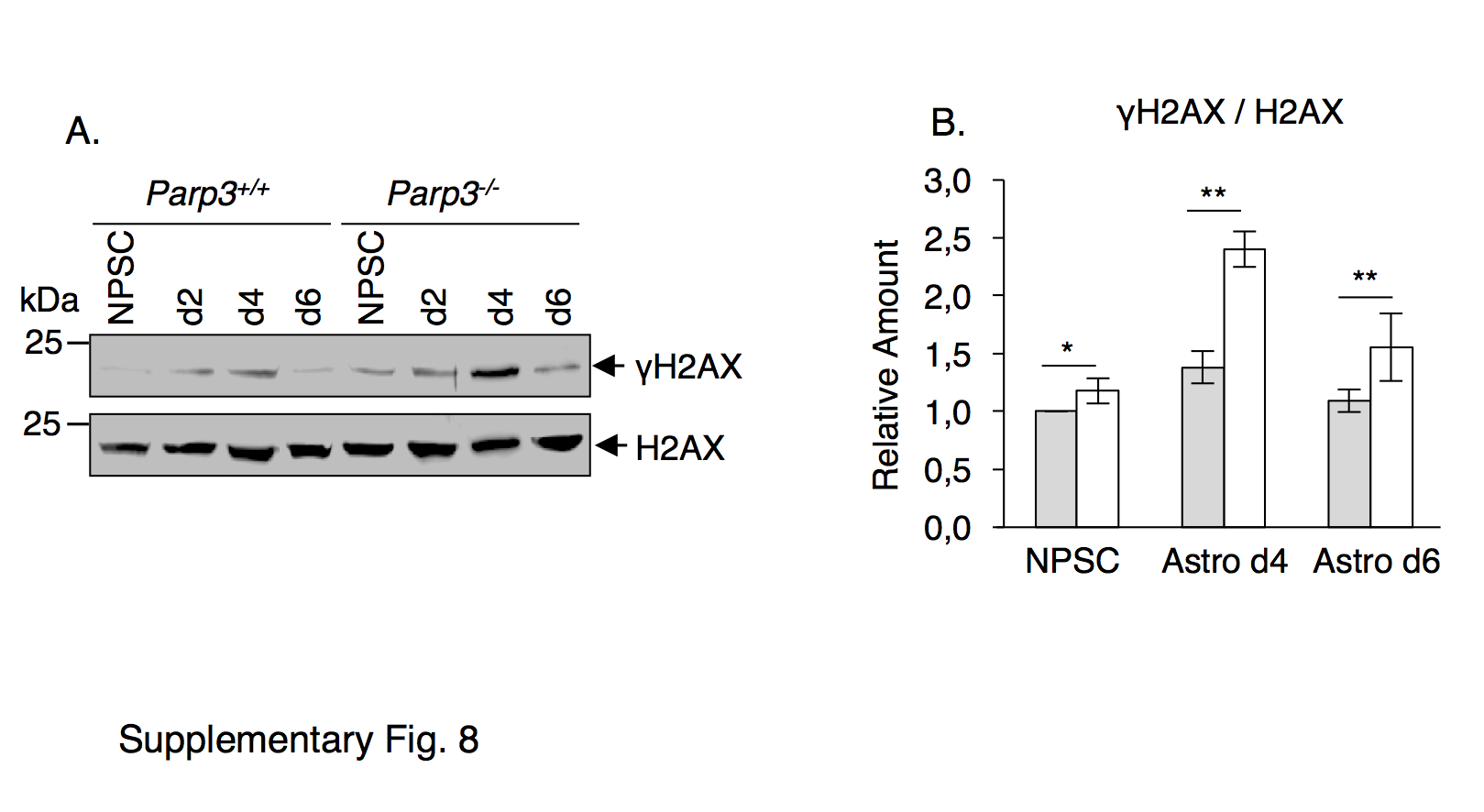

Supplement: Supplementary file 11 — Supplementary Figure 8 [file 41419_2020_3167_MOESM11_ESM.png]

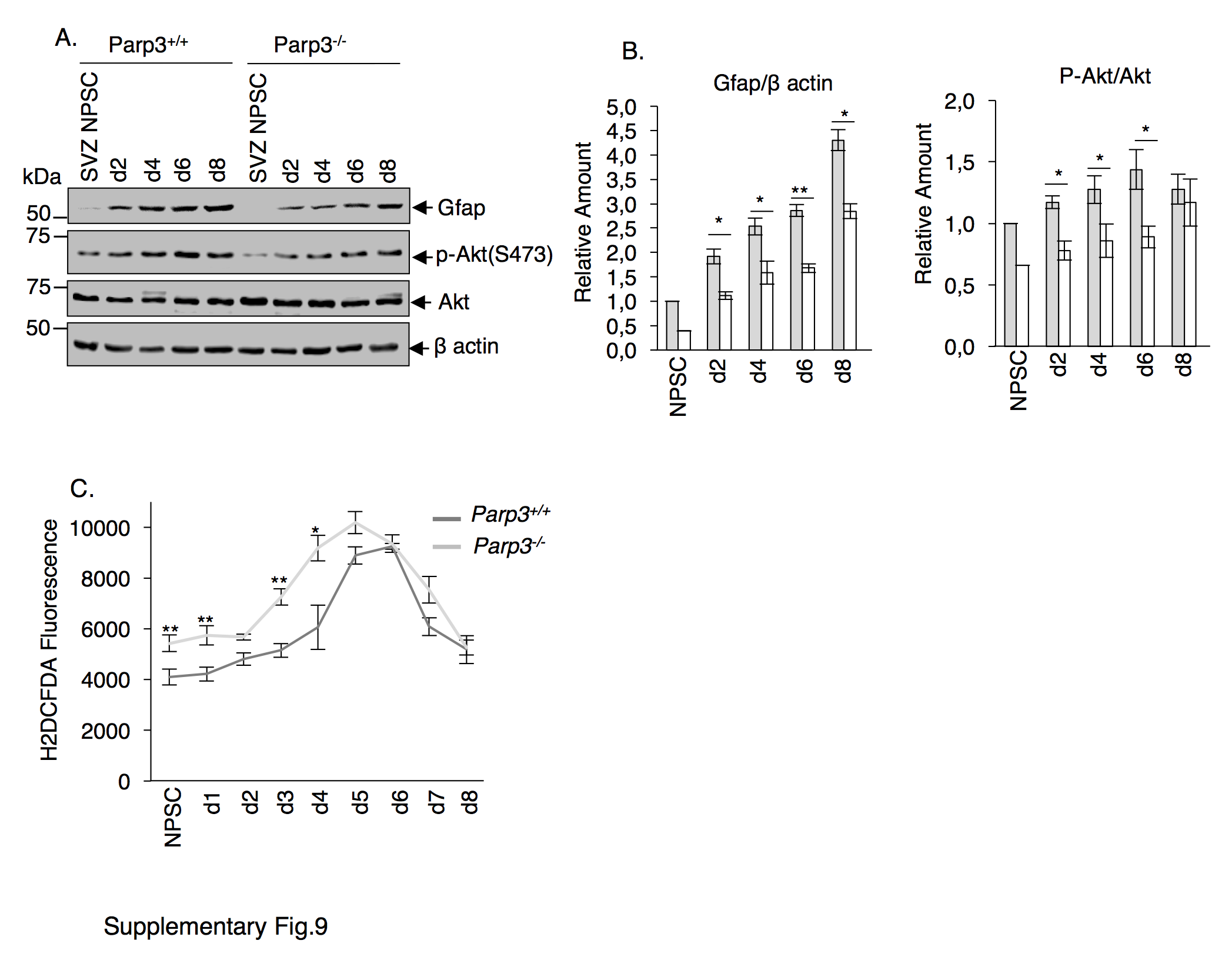

Supplement: Supplementary file 12 — Supplementary Figure 9 [file 41419_2020_3167_MOESM12_ESM.png]
