# Supplementary material for: Parp3 promotes astrocytic differentiation through a tight regulation of Nox4-induced ROS and mTorc2 activation
Source: Cell Death Dis. 2020 Nov 6;11(11):954. doi: 10.1038/s41419-020-03167-5 (PMC7648797; doi:10.1038/s41419-020-03167-5)
Supplement: Supplementary file 13 — Supplementary figure legends [file 41419_2020_3167_MOESM13_ESM.docx]

**Legends to Supplementary Figures 1-9**

**Supplementary Fig. 1** Parp3 is not required for neurosphere formation, proliferation and self-renewal. Related to Figure 1. **A**. Number of primary neurospheres obtained from *Parp3^+/+^* and *Parp3^-/-^* mice. Representative images of primary neurosphere cultures of both genotypes are shown. Values represent the means +/- s.d of three biological replicates and three independent clones. **B**. qPCR for the stemness markers *Nanog*, *Sox2* and *Oct4* of primary neurospheres from *Parp3^+/+^* and *Parp3^-/-^* mice in proliferation conditions. Data are expressed relative to *Gapdh.* Values represent the means +/- s.d. of ≥ 2 independent experiments and the three independent clones. **C.** Western blot analysis of the stemness markers Nanog and Sox2 relative to Gapdh in total extracts of 3 independent *Parp3^+/+^* and *Parp3^-/-^* NPSC cultures. **D**. Diameter of neurospheres formed at passage 2-4 in *Parp3^+/+^* and *Parp3^-/-^* cultures in proliferation conditions. Means are indicated in the boxplots as the red dots. Countings are from three independent experiments and three independent clones. **E**. Self-renewal capacity of neurospheres from *Parp3^+/+^* and *Parp3^-/-^* mice. Values represent the means +/- s.d. of three independent experiments and the three independent clones. f. Total number of NPSCs per neurosphere throughout passaging (P1-P4) in *Parp3^+/+^* and *Parp3^-/-^* cultures. Values represent the means +/- s.d. of three biological replicates and the three independent clones. **H**. Diameter of secondary neurospheres formed in *Parp3^+/+^* and *Parp3^-/-^* cultures in proliferation conditions. Means are indicated in the boxplots as the red dots. Countings are from three independent experiments and three independent clones.

**Supplementary Fig. 2** *Parp3^-/-^* astrocytes display an immature phenotype but viability is not impaired. Related to Figure 1. **A.** Bar graphs depict the % of Gfap+ cells (number of Gfap+/total number of Dapi+ cells) in Parp3^+/+^ and Parp3^-/-^ cultures throughout differentiation. Countings are from two independent experiments and two independent clones. Values represent the means +/- s.d. *<0,05, **P<0,01. **B.** Measurements of viability in *Parp3^+/+^* and *Parp3^-/-^* NPSC cultures and during astrocytic differentiation (d1-d7). Single NSPC cells were seeded at 4x10^4^ cells per well in 24- black-walled plates and processed for differentiation to astrocytes. At the indicated time points, cells were processed for calcein-AM staining (Interchim Flow probes) and calcein fluorescence production was measured using the Typhoon FLA 9500 Biomolecular Imager. Countings are from three independent experiments and three independent clones. Values represent the means +/- s.d. of three biological replicates.

**Supplementary Fig. 3** The absence of Parp3 sensitizes NPSCs to chemical-induced oxidative stress. Related to Figure 3. **A.** Neurosphere formation of *Parp3^+/+^* and *Parp3^-/-^* NPSCs 7 days after exposure to the indicated doses of Paraquat (PQ) for 1h compared to mock-treated cultures (UN). Values represent means +/-s.d. of three biological replicates and the three independent clones, **P<0,01, ***<0,0001. **B.** Neurosphere formation of *Parp3^+/+^* and *Parp3^-/-^* NPSCs 7 days after exposure to the indicated doses of Menadione (Menad.) for 1h compared to mock-treated cultures (UN). Values represent means +/-s.d. of three biological replicates and the three independent clones, **P<0,01. **C.** Neurosphere formation of *Parp3^+/+^* and *Parp3^-/-^* NPSCs 7 days after exposure to the indicated doses of H_2_O_2_ for 1h compared to mock-treated cultures (UN). Values represent means +/-s.d. of three biological replicates and the three independent clones, *P<0,05, **P<0,01. **D**. Representative images of *Parp3^+/+^* and *Parp3^-/-^* neurospheres 7 days after exposure to Paraquat (PQ), Menadione (Menad.) or H_2_O_2_. **E.** Diameter of neurospheres formed in *Parp3^+/+^* and *Parp3^-/-^* cultures 7 days upon exposure to H_2_O_2_, Paraquat (PQ) or Menadione (Menad.) for 1h compared to untreated cultures (UN). Means are indicated in the boxplots as the red dots. Countings are from three independent experiments and three independent clones. ****P<0,0001, ns, non-significant.

**Supplementary Fig. 4** The absence of Parp3 has no impact on the expression level of Duox1. Related to Figure 4. The bar graph depicts the relative fold increase of Duox1 levels in astrocytes relative to NPSCs in the *Parp3^+/+^* and *Parp3^-/-^* genotypes. Values represent means +/- s.d. of three independent experiments and two independent clones.

**Supplementary Fig. 5** Impaired mTorc2 signaling in Parp3-deficient cells is caused by reduced expression of Rictor but the expression levels of mSin1 and mLST8 are not affected. Related to Figure 5. Bar graphs depict the relative signal intensities of Rictor versus a-tubulin, mSin1 versus a-tubulin and mLST8 versus a-tubulin measured in the *Parp3^+/+^* and *Parp3^-/-^* genotypes and in three independent experiments using Image J. Mean values +/- s.d. are indicated. *<0,05, **P<0,01, ***P<0,001.

**Supplementary Fig. 6** Parp3 is involved in cell response to hypoxia. Related to Figure 7. **A.** Neurosphere formation of *Parp3^+/+^* and *Parp3^-/-^* NPSCs 7 days after exposure to the hypoxia mimetics CoCl_2_ and DFM for 48h compared to untreated cultures (UN). Values represent means +/- s.d. of three biological replicates and three independent NPSCs isolations. **P<0,01, ***P<0,001. **B.** Western blot analysis for the expression of Hif1a relative to b actin as loading control in *Parp3^+/+^* versus the *Parp3^-/-^* NPSCs either untreated (UN) or treated with the hypoxia mimetics CoCl_2_ and DFM.

**Supplementary Fig. 7** Parp3-catalysed ADP-ribosylation is temporarily induced by CoCl2. Related to figure 7. Western blot analysis of ADP-ribose formation in *Parp3^+/+^* and *Parp3^-/-^* NPSCs at the indicated time points upon exposure to CoCl_2_ with or without PARP inhibitor (Olaparib, 100 nM) and in the untreated (UN) cells. The gradual increase in CoCl2-induced ADP-ribosylation is temporarily reduced in Parp3-deficient NPSCs (T12h) suggesting a time-controlled induction of Parp3-catalysed ADP-ribosylation. The additional Olaparib-induced inhibition of ADP-ribosylation in the Parp3^-/-^ cells suggest that the residual ADP-ribose products can be due to PARP1 and/or PARP2 activity.

**Supplementary Fig. 8** Parp3-deficient NPSC and astrocytes display enhanced genome instability. Related to figure 4. **A.** Western blot analysis for gH2AX and H2AX in proliferating NPSCs and throughout differentiation to astrocytes in acid extracts from *Parp3^+/+^* and *Parp3^-/-^* cultures. **B.** The bar graph depicts the relative signal intensities of gH2AX relative to H2AX in *Parp3^+/+^* and *Parp3^-/-^* NPSC and astrocytes using Image J. Values represent means +/- s.d of three independent experiments and two independent clones, *< 0,05; **P<0,01.

**Supplementary Fig. 9** The absence of Parp3 compromizes p-Akt(S473), astrocytic differentiation, and ROS production in NPSC isolated from the subventricular zone (SVZ). Related to figures 1, 3 and 5. **A**. Western blot analysis for the levels of p-Akt (S473), Akt, Gfap and b actin as loading control in *Parp3^+/+^* and *Parp3^-/-^* SVZ-isolated NPSCs and throughout their differentiation to astrocytes (d2-d8). **B**. Bar graphs depict the relative signal intensities of p-Akt versus Akt, and Gfap versus b actin measured in three independent experiments using Image J. Mean values +/- s.d. are indicated. *<0,05, **P<0,01. **C**. Measurement of total ROS production in *Parp3^+/+^* versus *Parp3^-/-^* SVZ-isolated NPSCs and throughout astrocyte differentiation (d1-d8). Values represent means +/- s.d. of three biological replicates. *P<0,05, **P<0,01.
